# Underpinning hierarchical anisotropic structure formation in soy protein high-moisture extrudates by Magnetic Resonance Imaging^☆^

**DOI:** 10.1016/j.crfs.2026.101575

**Published:** 2026-09-26

**Authors:** Sam A. Kuijpers, Martijn I. Gobes, Morwarid Mayar, Frank J. Vergeldt, Johannes Hohlbein, Thom Huppertz, John P.M. van Duynhoven, Camilla Terenzi

**Affiliations:** a Laboratory of Biophysics, Wageningen University & Research, Stippeneng 4, 6708 WE Wageningen, The Netherlands; b Microscopy Research Facility, Wageningen University & Research, Stippeneng 4, 6708 WE Wageningen, The Netherlands; c FrieslandCampina, Stationsplein 4, 3818 LE Amersfoort, The Netherlands; d Unilever Global Foods Innovation Centre, Bronland 14, 6708 WH Wageningen, The Netherlands

**Keywords:** Texturised protein, Structure formation, High-moisture extrusion, Diffusion Tensor Imaging

## Abstract

Plant-based meat alternatives are fibrous anisotropic food protein products that mimic the texture and palatability of animal-based foods. The main industrially scalable production technique for manufacturing such structured protein materials is high-moisture extrusion, which involves the thermo-mechanical processing, and subsequent cooling, of a hot protein melt in the presence of shear and temperature gradients. Yet, the complex mechanisms governing fibrous structure formation in plant-protein extrudates are poorly characterised, in turn limiting the rational design and optimisation of the extrusion process. In the present work, we aimed at characterising the hierarchical nature of anisotropic structures in pH-shifted high-moisture extrudates of soy protein concentrate (SPC) by Magnetic Resonance Imaging (MRI). $T_{2}$-weighted MRI data, and corresponding Rotated Fourier Transform (RFT) analysis results, showed phase-separated protein-rich (PR) and water-rich (WR) lamellae at the 10^2^μm length scale. Chemical Exchange Saturation Transfer (CEST) MRI measurements showed the presence of dissolved proteins in the WR regions. Diffusion Tensor (DT)-MRI, and resulting Principal Diffusion Direction (PDD) maps, probe the hindrance of water self-diffusion down to the 10^-1^μm length scale and, here, revealed the presence of an underlying anisotropic structure in the PR lamellae. The latter finding was confirmed by Confocal Laser Scanning Microscopy (CLSM) measurements. The detailed features of RFT and PDD(DT) maps of these extrudates could be reproduced by simulating the effect of the cross-sectional cooling die geometry. Additionally, we found that the presence of structural order at the smallest length scale is a prerequisite for observing water diffusional anisotropy. We foresee that, in the future, the developed measurement methods will be deployed in the design and characterisation of protein-based products with a fibrous texture.

## Highlights

• SPC extrudates display a hierarchical structure at the 10^-1^ – 10^2^ μm length scales.
• $T_{2}$-weighted spin echo MRI quantifies anisotropy in SPC extrudates at the 10^2^ μm-scale.
• Diffusion Tensor MRI probes anisotropy in SPC extrudates down to the 10^-1^ μm-scale.

## Graphical abstract

**Figure:**
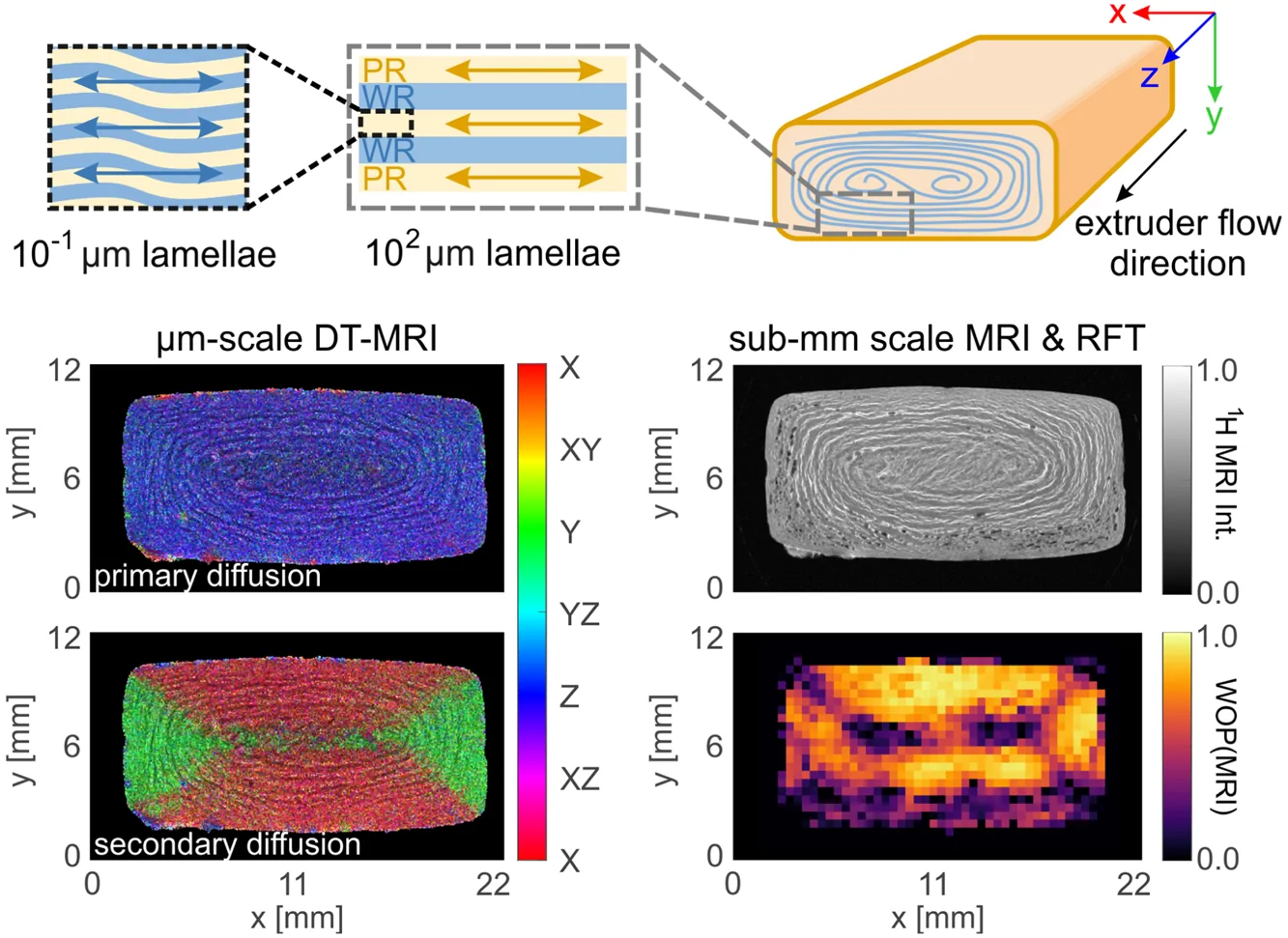

## 1 Introduction

Plant-based meat alternatives are textured protein products that provide consumers with a healthier and more sustainable alternative to animal meat, whilst offering a similar product palatability (Aiking and de Boer, 2020, Hoek et al., 2021, Collier et al., 2021). These products are commonly produced using High-Moisture Extrusion (HME) of commercially available plant-protein isolates, concentrates or flours, obtained mostly from soy or pea (Wang et al., 2023, Akdogan, 1999). HME involves a mixing and hydration step, followed by thermo-mechanical processing of the resulting protein dough inside the barrel section of the extruder (Akdogan, 1999). The high temperature and shear forces imparted on the protein dough during extrusion, and subsequent cooling in a cooling die, give rise to a meat-like fibrous texture. The key to design such protein textures is in understanding the relationship with the underlying hierarchical anisotropic structure (van der Sman and van der Goot, 2023, Kuijpers et al., 2025, Garina et al., 2026). A major determinant of anisotropic structure formation is shear, whose impact takes place at multiple length scales. In the extruder barrel section, insoluble carbohydrate fibres align in the continuous protein melt phase. Within this phase, also protein fibrils undergo alignment at the nm-scale (Guan et al., 2026, Garina et al., 2026). Subsequently, during cooling under shear inside the cooling die, the protein melt separates into protein-rich (PR) and water-rich (WR) domains at the sub-mm scale (Kuijpers et al., 2024). This anisotropic structure formation can be directed by tuning thermo-mechanical processing conditions (Pietsch et al., 2019), processing aids (Kuijpers et al., 2025, Li et al., 2026), protein source (Wang et al., 2023) and fibre content (Li et al., 2024, Deng et al., 2023). In this work, pH-shifting of extrusion of soy protein concentrate (SPC) is utilised to induce changes in structure formation at multiple length-scales (Kuijpers et al., 2025, Li et al., 2026).

To characterise such phase-separated anisotropic structured materials at the multiple length scales involved, an array of measuring modalities is required. For studies of protein networks, polarised fluorescence spectroscopy (Patel et al., 2025) can probe anisotropy at the molecular length scale. Small angle scattering techniques, either using a neutron or X-ray beamline, provide nm-scale insights into the building blocks that make up the anisotropic protein network (Garina et al., 2024, Kuijpers et al., 2025). Diffuse light reflectance provides a mm-scale average of sub-μm scale structural anisotropy (Garina et al., 2026, Van Beers et al., 2017). Confocal Laser Scanning Microscopy (CLSM) has been used to visualise the μm-scale structural anisotropy in HM extrudates of soy proteins (Li et al., 2026, Deng et al., 2023). X-ray tomography under cryogenic conditions has been used to spatially resolve the phase-separated sub-mm scale anisotropic structure of HM extrudates (Nieuwland et al., 2023, Li et al., 2026). Magnetic Resonance Imaging (MRI) has been used for the same purpose, but does not require cryogenic conditions (Kuijpers et al., 2024). Recently, Rotated Fourier Transform (RFT) analysis has been introduced to quantify structural anisotropy in two-dimensional images at multiple length scales (Gobes et al., 2025b). In the present work, we set out to quantify anisotropic structure in pH-shifted extrudates down to the 10^−1^ μm and 10^2^ μm length scales, respectively by CLSM and $T_{2}$-weighted MRI. Additionally, to probe structural order at the smaller length scales by MRI, we used the Diffusion Tensor (DT-)MRI (Basser et al., 1994). In food science, DT-MRI has been used to assess the quality of muscle fibres in meat (Bonny and Renou, 2002) and in seafood (Anderssen et al., 2022). DT-MRI uses Pulsed Field Gradients (PFGs) along the three Cartesian directions, applied prior to the MRI signal acquisition, to encode for the molecular self-diffusion, *e.g.* of water, and linear combinations of these PFGs to probe the self-diffusion anisotropy. DT-MRI measurements using six, or more, different diffusion encoding directions can be used to calculate a diffusion tensor for each voxel, providing both the magnitude and directionality of the self-diffusion (Basser et al., 1994, Kingsley, 2006a, Kingsley, 2006b, Kingsley, 2006c). In this work, we aimed at unravelling the factors governing water self-diffusion in SPC extrudates and, thus, we additionally used Chemical Exchange Saturation Transfer (CEST) MRI (Ward et al., 2000, van Zijl and Yadav, 2011) to assess the composition of the PR and WR domains.

## 2 Materials and methods

### 2.1 Materials

Commercial SPC (Arcon SJ® IP, 93% dry matter with 69% protein in dry base, 4% fat, 19% dietary fibre and carbohydrates and 2% ash), from Solae (St. Louis, MO, USA) was used in this work. The acidity and alkalinity of the water feed were adjusted by HCl and NaOH (Merck, Germany), respectively. Commercially manufactured high-moisture mozzarella was purchased from a local retailer.

### 2.2 High-moisture extrusion

Extrusion trials were performed as described in our previous works (Kuijpers et al., 2025, Kuijpers et al., 2024). A co-rotating twin-screw extruder (XTS 19, Xtrutech, UK) with a screw diameter of 19 mm and length-to-diameter (L/D) ratio of 25 was used. The extruder was equipped with a rectangular cooling die (17 mm (width) × 7 mm (height) × 300 mm (length), with rounded corners of radius 0.5 mm) and coupled to the extruder through a die adapter. The extruder barrel included four consecutive temperature-controlled zones, respectively heated to 80 °C, 120 °C, 160 °C, and 135 °C in zones 1 to 4. The barrel exit temperature was set to 135 °C. The water used to cool the cooling die was maintained at a temperature of 65 °C. Extrusion was performed using tap water to obtain extrudate at pH 7.0, and extrudates shifted to pH 5.0 and 7.8 were obtained by changing the liquid feed respectively to 0.35 M HCl or 0.05 M NaOH as described in Ellwanger et al. (2024) and Kuijpers et al. (2025). The extrusion trials were run at a screw speed of 350 rpm and a moisture content of (60 ± 0.5) %. Extrudate samples were collected from the cooling die outlet after reaching a steady-state, as indicated by constant process responses. The samples were put in a zip-lock bag, immediately cooled in ice-water and, subsequently, frozen and stored at −18 °C. Freezing is an integral part of structure formation in protein extrudates and has previously been demonstrated to enhance the existing lamellar anisotropic structure and improve MRI contrast between lamellae (Kuijpers et al., 2024).

### 2.3 Sampling reference system

In the present work, combinations of Cartesian positions, *i.e.* X, Y and Z, and anatomical planes, *i.e.* axial, sagittal and coronal, are used to describe the position and orientation of the imaging planes within the sample with respect to the extrusion direction. MRI imaging slices will refer to anatomical planes, namely, axial (XY) and sagittal (YZ). In Fig. 1, two such planes, namely the sagittal and axial planes, are represented with respect to a schematic extrudate sample. The X (red) and Y-axes (green) refer to the width and height of the sample, and the Z-axis (blue) corresponds to the flow direction in the extruder. Principal Diffusion Direction (PDD) maps, calculated from Diffusion Tensor (DT-)MRI data, encode diffusion directions with respect to the Cartesian axes using the colour scheme shown in Fig. 1. The location within an intact extrudate sample, where the sample was cut for CEST measurements, is shown by the green volume in the latter figure. The PR and WR domains are respectively illustrated by yellow and blue (Kuijpers et al., 2024).

**Fig. 1:**
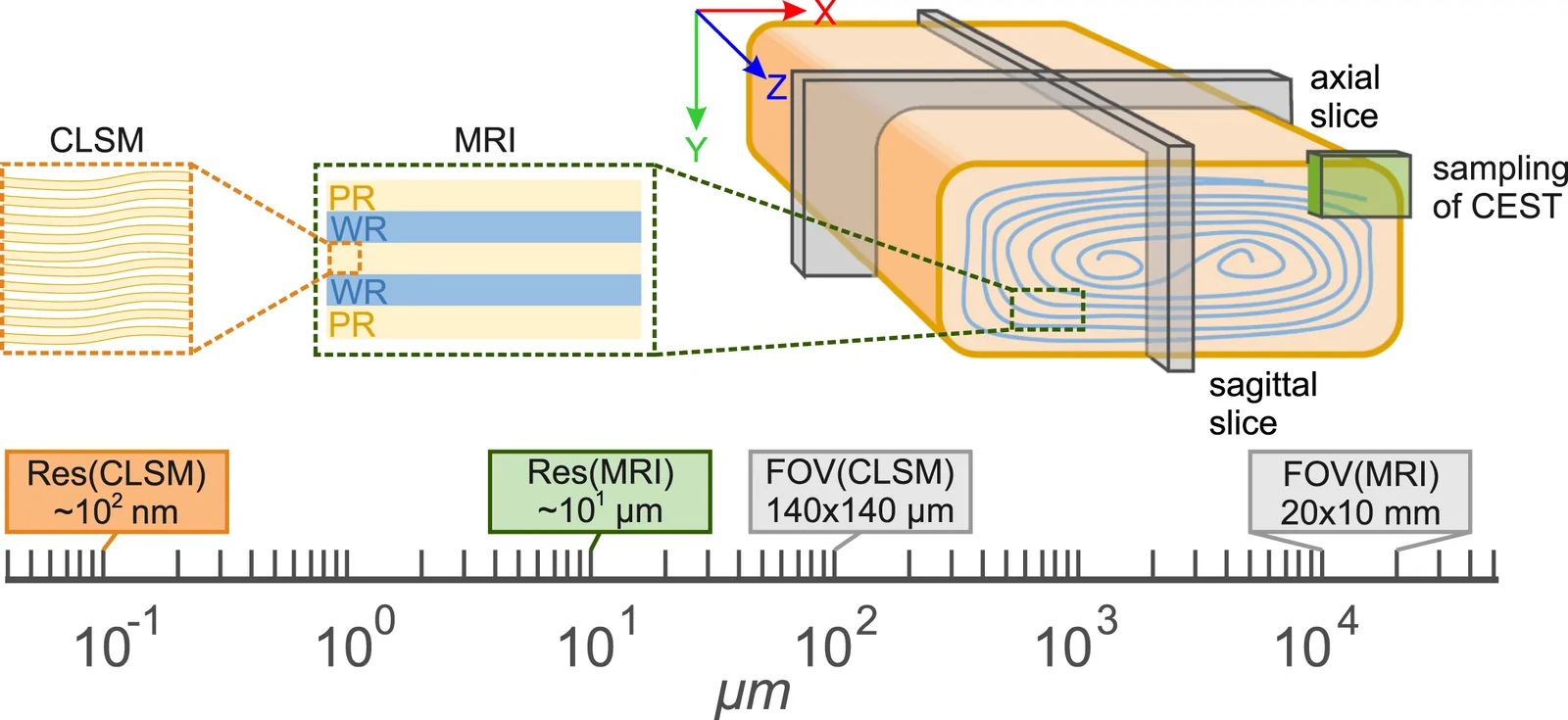
A schematic representation of an extrudate sample showing the MRI imaging planes with respect to the Cartesian axes. The extruder flow direction is along the Z-axis. The slice thickness for MRI is 0.5 mm for $T_{2}$-weighted images and 1.0 mm for DT-MRI images. The green box indicates the sample cut for CEST MRI measurements at 28.2 T and were performed using a slice thickness of 100μm at an in-plane MRI resolution of 50μm (Callaghan and Eccles, 1988). CLSM in combination with an Airyscan detector has a theoretical resolution of 100 nm, below the limit imposed by the diffraction of light (Hohlbein, 2021).

### 2.4 Magnetic Resonance Imaging (MRI)

#### 2.4.1 MRI sample preparation

A common part of the production process of meat alternative materials is marination. Thus, to mimic this post-processing, and to enhance the signal intensity and contrast between lamellae during MRI measurements, samples were swollen with water (Kuijpers et al., 2024). For ^1^H MRI CEST measurements, a 2 mm slice was cut from a frozen extrudate ribbon of approximately 1 cm long (Fig. 1) along the extruder flow direction (see Fig. S1.1 in SI). For water swelling, the thawed sample was placed inside a 5 mm NMR tube containing demineralised water. After 15 min. of swelling, the tube containing sample and water was placed in a vacuum desiccator for 20 min to remove residual air bubbles. The sample was left in demineralised water to continue swelling overnight for ca. 18 h. Prior to measuring, a Pasteur pipette was used to remove the excess water around the sample inside the 5 mm NMR tube and the tube was sealed with parafilm to prevent water evaporation and sample contamination during MRI measurements.

For Rapid Acquisition with Refocused Echoes (RARE) and DT-MRI measurements, a sample of approximately 3 cm length was cut from a frozen extrudate ribbon and placed in a 50 mL tube. This sample was left in demineralised water to swell overnight for ca. 18 h at room temperature. The volume ratio of water to sample was 15:1. Air bubbles were removed from the sample by placing the tube in a vacuum desiccator for 20 min. After swelling, excess moisture was removed from the outer surface of the extrudate using a paper towel. The sample was placed in a 25 mm NMR tube, which was sealed with parafilm to prevent water evaporation and sample contamination during MRI measurements.

For RARE and DT-MRI of mozzarella cheese, a cube of sample was cut and placed in a 25-mm MRI tube containing a NaCl solution matching the salt content on the packaging, to prevent drying during measurements.

#### 2.4.2 MRI imaging procedure

^1^H CEST MRI measurements (Ward et al., 2000) were performed using a 28.2 T (1.2 GHz for ^1^H) narrow-bore spectrometer (Ascend 1.2 GHz, Avance Neo, Bruker, Germany) equipped with a 2.85 T/m gradient system (Micro 5, Bruker, Germany) and a 5 mm micro-imaging probe (Iprobe, Bruker, Germany). The measurements were conducted in Paravision 360 version 3.3. In the CEST MRI pulse sequence, a saturation pulse was first applied, consisting of 10 hard pulses, each of length $t_{p}=100\;\mathrm{ms}$ and with inter-pulse delay $t_{delay}=10\;\mu s$, resulting in a total saturation time $T_{sat}=1\;s$ (Mayar et al., 2023). The amplitude of the saturation pulse, $B_{1}=1.5\;\mu T$, was optimised for detecting water-protein chemical exchange and relayed Nuclear Overhauser Enhancement (rNOE) effects in the CEST spectrum (Mayar et al., 2023, van Zijl and Yadav, 2011). The image readout was performed with a RARE sequence (Hennig et al., 1986) and a single MRI slice was excited at the centre of the sample in the axial plane with a thickness of 100μm (Fig. 1). The MRI images acquired after a saturation pulse, $S_{sat}$, were taken at varying frequency offsets, Δω. For the saturation pulse, 65 values were chosen between −10 and 10 ppm. From −10 to −6 ppm and 6 to 10 ppm, frequency offset values were placed 0.3 ppm apart. Between −4 ppm and 4 ppm, the resolution was increased to 0.1 ppm between frequency offsets values. In addition, two reference unsaturated MRI images, $S_{0}$, were acquired at Δω=± 450 ppm. The RARE imaging parameters were as follows: echo time $T_{E}=5\;\mathrm{ms}$; repetition time $T_{R}=3.6\;s$; RARE-factor of 2; 4 signal averages; Field-Of-View (FOV) of 5 × 5 mm^2^; MRI resolution of 50 × 50 × 100μm^3^. This resulted in a measurement duration of about 12 min per Δω value, and of about 13 h for the full CEST MRI measurement.

^1^H DT-MRI and RARE measurements were performed using a 14 T (600 MHz for ^1^H) wide-bore spectrometer (Ascend 600WB, Avance NEO, Bruker, Germany) equipped with a 1.5 T/m imaging gradient system (Micro 2.5, Bruker, Germany) and a 25 mm quadrature ^1^H r.f. probe (MicWB40/025 QTR, Bruker, Germany). The measurements were performed using ParaVision 360 version 3.6 (PV-360.3.6). $T_{1}$- and $T_{2}$-weighted MRI images of SPC extrudates were acquired using the RARE sequence in both the axial and sagittal orientation (Fig. 1). Acquisition parameters were set as follows: $T_{E}=10\;\mathrm{ms}$; $T_{R}=1\;s$; RARE-factor of 2; 4 signal averages and 4 imaging slices of 0.5 mm thick, were selected at the centre of the sample and inter-spaced by 1 mm; FOV of 22.5 × 12.5 mm^2^ or 25 × 12.5 mm^2^, respectively for axial or sagittal images; MRI resolution of 39 × 39 × 500μm^3^, with a total measurement duration per image acquisition of 11 min for each orientation.

Water self-diffusion anisotropy measurements were performed using a stimulated-echo based DT-MRI pulse sequence (Basser et al., 1994). Pulsed field gradients (PFGs) are used to encode the water self-diffusion, with the amount of diffusion encoding being defined by $b={\left(\gamma \delta G\right)}^{2}\left(\Delta -\frac{\delta }{3}\right)$ in s/mm^2^. This parameter is determined by the gyromagnetic ratio of hydrogen, γ, the strength of the diffusion encoding gradient, G, the duration of diffusion-encoding gradients, δ, and the self-diffusion encoding time Δ (Stejskal and Tanner, 1965). The stimulated-echo acquisition scheme was chosen in order to account for the short $T_{2}$ values, typically between 8 and 30 ms (Kuijpers et al., 2024), observed for the water-swollen SPC extrudates at 14 T in the presence of the adopted imaging gradients. Thus, to enable the use of long enough Δ values between consecutive PFG pulses, magnetisation was stored along the −z axis, where longitudinal relaxation takes place. The $T_{1}$ longitudinal relaxation constant is, for the investigated samples, typically between 500 and 1500 ms (Kuijpers et al., 2024). An optimal Δ value of 50 ms was chosen to observe the hindered water self-diffusion with sufficient Signal-to-Noise ratio (SNR > 40) for accurate fitting of the diffusion tensor (see Fig S1.2 in SI), while avoiding excessive $T_{1}$ saturation effects. DT-MRI acquisition parameters were set as follows: $T_{E}=13\;\mathrm{ms}$; $T_{R}=1\;s$; δ=2ms; Δ=50ms; 8 signal averages (see Fig S1.3 in SI) per axial imaging slice at the centre of the sample (Fig. 1); FOV of 22.5 × 12.5 mm^2^; MRI resolution of 52 × 52 × 1000μm^3^. In PV-360.3.6, multiple so-called ‘experimental shells’ can be defined to vary both the number of diffusion encoding directions and the diffusion-weighing b-values, within one measurement. For each DT-MRI acquisition, a two-shell acquisition scheme was chosen, with directions and diffusion encoding values determined as described in Kingsley, 2006a, Kingsley, 2006b, Kingsley, 2006c. The first shell consisted of 8 reference MRI images without use of diffusion-encoding gradients, hereafter called $A_{0}$-images. Due to the gradients required, either for imaging or for signal crushing between subsequent acquisitions, even in the absence of diffusion encoding gradients, an effective b-value, namely $b_{\text{eff}}=120\text{s}/{\text{mm}}^{2}$, was produced for the acquisition of the $A_{0}$-images. $A_{0}$-images were manually set as the first experimental shell with a $b_{\text{eff}}$ of 120 s/mm^2^ (See Fig S1.4 in SI). The second experimental shell contains the different diffusion encoding gradient combinations which enable probing diffusion in different directions. The optimal number of directions was determined to be 36 (See Fig S1.5 in SI) and the probed diffusion directions are calculated in Paravision using a model that simulates opposed point charges (Jones et al., 1999, Kingsley, 2006b). Here, 36 unit vectors were distributed evenly on a half-sphere due to the simulated repelling charges between these vectors. The projections of each vector on the Cartesian axes are used to calculate the required PFG strength of the read, phase and slice-selective gradients. These 36 diffusion encoding directions were acquired using a $b_{\text{eff}}$ of 1000 s/mm^2^. The resulting total measurement duration per sample was 23.5 h.

DT-MRI tensor calculations are known to be sensitive to noise, resulting in partly inaccurate or negative-valued tensor elements (Kingsley, 2006c, Jones and Basser, 2004). Thus, the imaging parameters described above were determined performing a series of measurements upon linearly varying either Δ (Fig S1.2 in SI), or the number of averages (Fig S1.3 in SI), or the number of diffusion encoding directions (Fig S1.5 in SI), to assess the impact of these parameters on the SNR. The criterion these acquisition parameters were weighted against was the relative amount of negative-valued tensor elements with respect to the average SNR. If less than 5% of tensors contained negative elements, the parameter was chosen to be optimal.

RARE image acquisition of the mozzarella sample was performed using the following parameters: $T_{E}=15\;\mathrm{ms}$, $T_{R}=1\;s$, 2 signal averages, MRI resolution = 78 × 78 × 1000μm^3^. The DT-MRI STE measurement was acquired using: $T_{E}=12.5\;\mathrm{ms}$, $T_{R}=1\;s$, 8 signal averages, MRI resolution = 78 × 78 × $1000\;\mu m^{3}$, 8 $A_{0}$-images at $b_{\text{eff}}=120\;s$/mm^2^ and 36 images at $b_{\text{eff}}=1000\;s$/mm^2^.

#### 2.4.3 MRI data analysis

^1^H MRI data processing and image analysis was performed in MATLAB R2022b using the pvmatlab package by Bruker for extracting raw MRI data from PV-360.3.6. In-house scripts for automated data processing were written in MATLAB R2022b using functions from the Image Processing Toolbox version 11.6 and Curve Fitting Toolbox version 3.8.

CEST MRI data was corrected for $B_{0}$ inhomogeneities by performing a per-voxel fitting of the $S_{sat}$ values as a function of Δω using a smoothing-spline interpolation. The water frequency shift was defined as the Δω value corresponding to the position of the minimum in the fitted signal intensity curve (resolution 0.01 ppm). A $\delta B_{0}$-map, representing the $B_{0}$ inhomogeneity, was constructed per-voxel by correcting the position of the minimum of the measured signal intensity dispersion curve to Δω=0ppm (Stancanello et al., 2008). CEST spectra were constructed per voxel by normalising the $B_{0}$-corrected $S_{sat}$ images by the reference unsaturated image, $S_{0}$. Intensity thresholding of the $S_{0}$ image at Δω=450ppm was conducted using Otsu’s method with three threshold values (Otsu, 1979). Voxels with intensities above the third, and below the second, threshold value were respectively attributed to the WR and PR regions. CEST spectra were averaged over either the water- or protein-rich voxels to determine the spatial distribution of proteins over the two phases.

Calculations of the diffusion tensor, average diffusivity ($D_{av}$) and fractional anisotropy (FA) from the diffusion-weighted images, were performed in MATLAB using the procedures described in Kingsley, 2006a, Kingsley, 2006b, Kingsley, 2006c. The $D_{av}$ and FA are calculated using linear combinations of the three tensor eigenvalues: $\lambda_{1}$, $\lambda_{2}$ and $\lambda_{3}$, respectively corresponding to the major, intermediate and minor diffusion components as follows:

(1) $$D_{av}=\left(\lambda_{1}+\lambda_{2}+\lambda_{3}\right)/3,$$

(2) $$FA=\sqrt{\frac{3\left[{\left(\lambda_{1}-D_{av}\right)}^{2}+{\left(\lambda_{2}-D_{av}\right)}^{2}+{\left(\lambda_{3}-D_{av}\right)}^{2}\right]}{2\left(\lambda_{1}^{2}+\lambda_{2}^{2}+\lambda_{3}^{2}\right)}}.$$

The eigenvalues are conventionally sorted by their magnitude, $\lambda_{1}$ $>\lambda_{2}$ $>\lambda_{3}$. However, this method may introduce statistical artifacts in the eigenvalue distribution caused by background noise in the acquired data affecting the tensor calculation. The result is a bias in the mean eigenvalues within a homogeneous region of interest (ROI), especially in more isotropic regions (Basser and Pajevic, 2000). To correct for this, after having sorted the eigenvalues by their magnitude, the inter-voxel overlap between two diffusion tensors is calculated as described by (Basser and Pajevic, 2000):

(3) $$C_{t}=\frac{\overset{3}{\underset{i=1}{\sum }}\lambda_{i}\lambda_{i}^{\prime }{\left(\epsilon_{i}\cdot \epsilon_{i}^{\prime }\right)}^{2}}{\overset{3}{\underset{i=1}{\sum }}\lambda_{i}\lambda_{i}^{\prime }}.$$

Here, $0<C_{t}\leq 1$, with 0 indicating no overlap and 1 indicating complete overlap of the tensors. The sum of each eigenvalue-eigenvector (ϵ) pair is compared between the voxel of interest ($\lambda_{i}$, $\epsilon_{i}$) and a reference tensor ($\lambda_{i}^{\prime }$, $\epsilon_{i}^{\prime }$). This reference tensor is taken as the mean diffusion tensor of an ROI around the voxel of interest. The optimal size of the ROI was found to be a 9 × 9 voxel area, defined as the area where a further increase in the ROI size does not appreciably alter the mean eigenvalue distribution (See Fig. S1.6 in SI). For each voxel in the image, $C_{t}$ is calculated for the six unique possible eigenvalues-sorting combinations and, thus, the sorting order with the highest overlap is chosen. From these ROI-sorted eigenvalues, PDD maps are created by encoding the angle of the eigenvectors of each eigenvalue for a Red, Green and Blue (RGB) value based on their direction (Makris et al., 1997). This results in three images corresponding to the three different eigenvalues of the tensor. The resulting RGB-values between 0 and 1 are weighted by their respective FA values, to highlight regions with high anisotropy. Tensors with one or more negative eigenvalues were omitted from the calculations of the diffusion coefficient, FA or PDD maps.

### 2.5 Confocal Laser Scanning Microscopy (CLSM)

#### 2.5.1 Sample preparation for CLSM

A frozen extrudate ribbon, of approximately 1 cm long, was thawed. A ∼0.5 mm thin slice was taken from the middle of the thawed ribbon (similar to taking a sagittal MRI image slice, see Fig. 1) using a razor blade. The sample was placed on a 125μm thick glass slide. A droplet of dye was placed on the sample and allowed to equilibrate with the sample for ca. 1 h. The dye consisted of Rhodamine B (RhB, Exciton, Lockbourne, USA) to stain proteins, and Calc-Fluor white (CFW, ID: 18909, 1 g/L with 0.5 g/L Evans blue, liquid form, Merck, Rahway, USA) to stain polysaccharides. A solution of RhB in ethanol was first prepared by dissolving 50 mg of RhB in 1.5 mL of 98% pure ethanol (Reagent Grade, Sigma-Aldrich, Germany). Then, 15μL of the RhB solution and 50μL of the liquid CFW solution were dissolved in 1.5 mL of PEG-200 to minimise the effect of sample swelling due to the due uptake (Gobes et al., 2025b).

#### 2.5.2 CLSM imaging procedure

CLSM imaging was performed on a Zeiss confocal microscope (Zeiss LSM 880, Zeiss, Germany) equipped with an Airyscan detector and Zeiss Plan-Apochromat 63x/1.4 NA DIC M27 Oil immersion objective (Zeiss, Germany). Hereafter, the CSLM-Airyscan combination will be referred to as CLSM. The Zeiss confocal used a 405 nm and 514 nm laser line, respectively at 4% and 5% laser power. The detector ranges are 410 – 509 nm for CFW and 531 – 703 nm for RhB. Three images were obtained per pH-shifted sample using a spatial resolution of 44 × 44 nm^2^ and a FOV of 140 × 140μm^2^.

### 2.6 Rotated Fourier Transform (RFT) analysis

RFT analysis was used to obtain quantitative vector field maps, displaying structural orientational order, from MRI and CLSM images, as described in full detail in Gobes et al. (2025b). ImageJ (FIJI, 64-bit, version 1.54j) was used with the RFT plugin version 0.1 installed to extract feature angles from square 51 pixel or 301 pixel image windows, respectively for MRI or CLSM images, and using an overlap setting of 75%. The optimal windows size is set to be roughly 5–10× the size of the feature of interest (Gobes et al., 2025b), respectively 5–12 pixels for MRI and 23–46 pixels for CLSM. An in-house developed Python routine was used to convert feature angles per window into 2D spatial maps of feature angle, relative angle intensity and Weighted Order Parameter (WOP) (Gobes et al., 2025b). The WOP maps presented in this work are acquired by analysing a 5 × 5 vector ROI per WOP value. To limit the effect of large intensity differences at the interface between sample and background, leading to incorrect assignment of the angle of alignment in the vector field, the initially masked background of the MRI images was changed from zero to the mean pixel intensity of each sample prior to RFT analysis. The 51 pixel image windows in MRI correspond to a size of 2 × 2 mm^2^ and the calculated WOP value averages information from a 4 × 4 mm^2^ region. Concurrently, the 301 pixel image windows in CLSM correspond to a size of 13 × 13μm^2^ and the corresponding WOP values averages information from a 26 × 26μm^2^ region.

### 2.7 Generation of synthetic axial MRI data for cooling die geometries

The cross-sectional cooling die geometry is most commonly rectangular with rounded corners. This geometry was also studied in this work. At pilot-scale HME conditions, sometimes these corners are completely rounded and create a so-called bullnose geometry (Garina et al., 2026). For the purpose of simulating the effect of significantly varying the cross-sectional cooling die geometries, we have considered two additional extreme cases of square-corner and elliptical geometries. The synthetic axial MRI data for each cooling die geometry was generated as follows. (i) A uniform high-intensity image, normalised to a value of 1, containing 1000 × 500 pixels in the XY-plane (Fig. 1) was created using the same aspect ratio (2:1) as in the axial MRI images. (ii) At the image centre, a zero-intensity circular outline was placed with a diameter of 25 pixels. (iii) The latter step was repeated for increasing circle diameters up to the full image size in the Y-dimension, while avoiding any overlap between circles. (iv) The image intensity values were inverted to create a zero-intensity background with high-intensity features, and the resulting image was saved in the tiff format. This process was repeated for ellipses with different ratio between the major and minor axes up to 2:1, as well as for rectangles with full rounded edges and sharp edges. Images were analysed by RFT (Gobes et al., 2025b) using a window size of 51 pixels and an overlap op 75%. Resulting vector field maps were converted to WOP and PDD maps as described in previous sections.

**Fig. 2:**
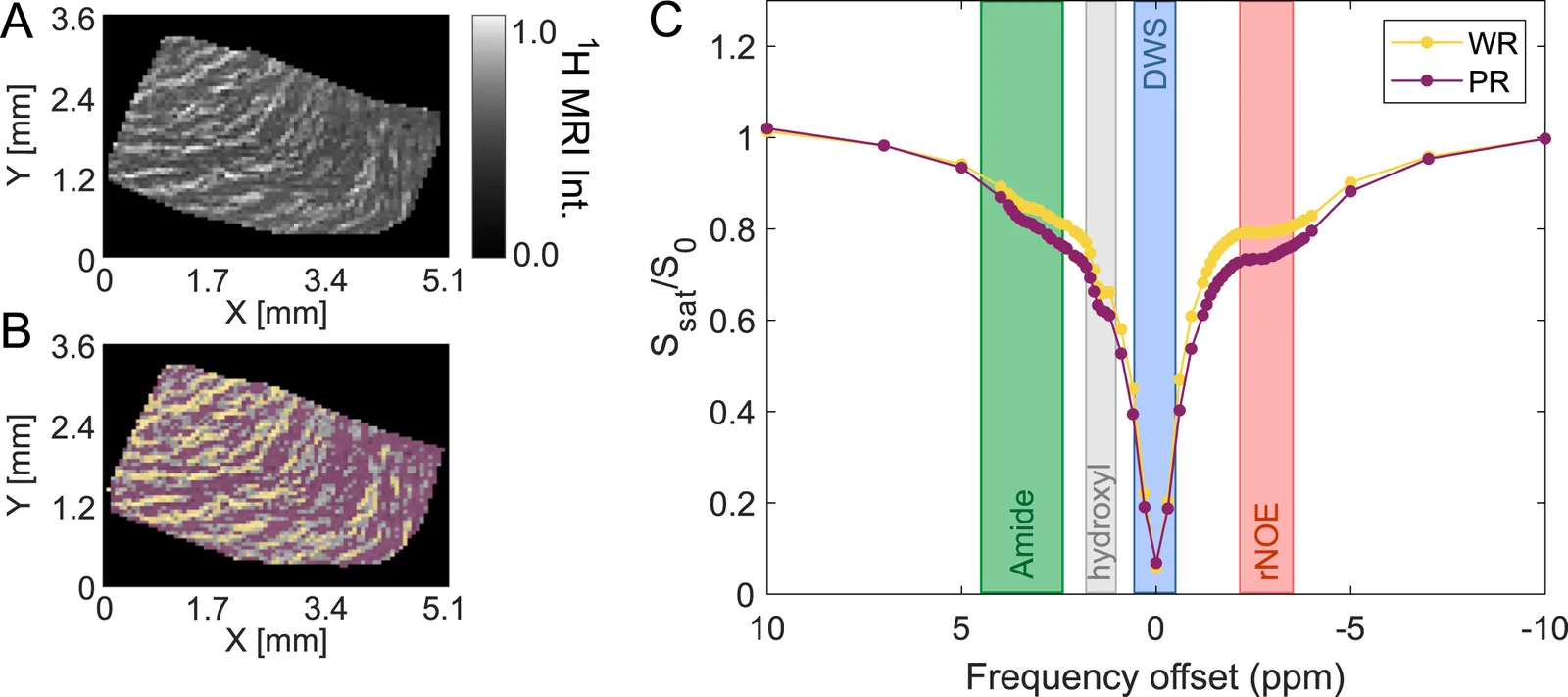
(A) Axial $T_{2}$-weighted ^1^H MRI image of a frozen, thawed and swollen SPC extrudate in which WR and PR regions respectively have higher and lower intensity. (B) Segmentation of the WR (yellow) and PR (purple) regions based on intensity thresholding with lower and upper threshold values of 0.23 – 0.45 for the PR phase and 0.59 – 1 for the WR phase. The regions in green (between 0.45 – 0.59) are considered to have partial volume effects between PR and WR. (C) ^1^H CEST spectrum of the WR and PR regions in the SPC extrudate.

## 3 Results and discussion

### 3.1 Assessment of solubilised protein fractions in WR regions

To aid the characterisation of the self-diffusion of water in the phase-separated structure of the SPC extrudates, the presence or absence of solubilised proteins in the WR phase was assessed using CEST MRI measurements performed at ultra-high field (28 T). At such condition, the high ^1^H MRI sensitivity enabled sufficient SNR for CEST MRI at near isotropic voxel sizes of 50 × 50 × 100μm^3^. The $T_{2}$-weighted MRI image (Fig. 2A) of the SPC extrudate shows a separation into high- and low-intensity voxels, respectively corresponding to the WR and PR phases (Kuijpers et al., 2024). These two phases were quantified by intensity thresholding using Otsu’s Method (Fig. 2B) (Otsu, 1979), respectively using the lower and upper threshold values of 0.23 – 0.45 for the PR phase and 0.59 – 1 for the WR phase. The WR phase made up 15.1% of the sample volume, whereas the PR phase was more dominant, constituting 51.7%. The remaining 33.2% of voxels likely still contained both WR and PR phases due to partial volume effects and were therefore omitted from the analysis of the CEST spectra.

The CEST spectra (Fig. 2C) of both the WR and PR phases show the amide (Δω=3.5ppm) and hydroxyl (Δω=1.3ppm) chemical exchange peaks at positive offsets with respect to the direct water saturation (DWS) peak. These peaks arise from ^1^H chemical exchange between mobile proteins or peptides and water. Additionally, a peak resulting from the relayed Nuclear Overhauser Enhancement (rNOE) can be observed at Δω = −3 ppm. The rNOE effect arises from a two-step process in which magnetisation of non-exchangeable protons is transferred via through-space dipolar coupling to neighbouring exchangeable protons and, subsequently, to water via ^1^H chemical exchange (Zhou et al., 2023). The amide and rNOE peak are not observed in the CEST spectrum of water and methylcellulose (see Fig. S2.1 in SI). The rNOE peak could be observed at the same Δω of −3 ppm in the CEST spectrum of a 1 wt% soy protein isolate (SPI) solution (see Fig. S2.1 in SI), indicating that the rNOE peak for the WR phase of the SPC extrudate resulted from the presence of protein and not from the hydroxyl groups of cellulose, the latter being present in SPC. The through-space dipolar coupling effect is characteristic of large, but mobile, proteins shown to generally have rotational correlation times of ∼100 ns (Jones et al., 2013, Zhou et al., 2023). Therefore, the rNOE peak indicates the presence of solubilised proteins in the WR phase. These features observed in the CEST spectra indicate the presence of proteins in both phases. The intensity of the peaks, and thus the concentration of proteins, is lower in the WR phase than in the PR phase. Low-intensity amide and rNOE peaks were observed in the CEST spectra of the WR phase of a swollen fresh SPC extrudate (see Fig. S2.2 in SI). This indicates that, even before freeze-thawing, the delaminated WR phase of SPC extrudates already contains solubilised proteins. Using this method, it cannot be excluded that proteins from the PR phase were solubilised into the WR phase during swelling. However, DT-MRI measurements performed in this work are on frozen, thawed and swollen extrudates which, have experienced the same post-processing steps as the sample used to acquire the CEST MRI data in Fig. 2C.

### 3.2 Anisotropic structure quantification at 10^−1^μm and 10^2^μm-scale

Fig. 3 shows the sagittal MRI images of pH-shifted SPC extrudates. In line with our previous MRI results on pH-shifted SPC extrudates (Kuijpers et al., 2025), anisotropic phase-separated lamellar structure is visible at pH 7.0 and 7.8. At pH 5.0, approaching the iso-electric point of SPC (pI ∼ 4.2) (Kuijpers et al., 2025), the phase-separated structure appears heterogeneously distributed. Concomitantly, at pH ≫ pI, here pH 7.8, the lamellar phase structure appears coarser than at pH 7.0 (Kuijpers et al., 2025). The WOP(MRI) maps follow a similar trend to our previous work (Kuijpers et al., 2025), with WOP(MRI) values being the lowest at pH 5.0, (0.4 ± 0.4) and (0.5 ± 0.4) respectively for Axial and Sagittal orientations, where no evidence of a uniform aligned phase-separated structure is observed along the extruder flow direction. At pH 7.0 and 7.8, the phase-separated lamellae are oriented along the flow direction, and the WOP(MRI) values are high throughout the sample. Here WOP(MRI) values are (0.7 ± 0.3) and (0.7 ± 0.2) respectively for pH 7.0 and 7.8 in the axial orientation and (0.9 ± 0.2) and (0.8 ± 0.2) in the sagittal orientation. For both orientations, ANOVA of the WOP distributions yielded p<0.01. WOP(MRI) distributions are shown in Fig S2.3 in the SI. Towards the middle of the sample, at Y = 6 mm (cyan arrow), lower WOP(MRI)-values are obtained, reflecting the local change in lamellae orientation (Gobes et al., 2025b). Additionally, regions with air bubbles exhibit lower structural order. Similar features are observed for the axial image orientation presented in Fig. 3. In the centre of the axial images, a perturbation of the lamellar structure can be observed, which indicates that the hot protein melt entered the cooling die with a memory of the co-rotating screws in the extruder (Garina et al., 2026). As shown in Fig. 3 and previous work (Kuijpers et al., 2025, Garina et al., 2026, Gobes et al., 2025b), RFT is able to quantify anisotropic structure in MRI images. However, due to the requirement of several voxels per vector calculation and subsequently several vectors per calculated WOP(MRI) value, the smallest assessable length scale is more than ten times the initial voxel size. To analyse the 10^−1^ μm-scale structure of pH-shifted SPC extrudates, CLSM images were acquired for the same pH-shifted samples as for the previous MRI measurements. The bottom half of Fig. 3 shows the CLSM images of pH 5.0, 7.0 and 7.8 samples, and their respective WOP(CLSM) maps. In the MRI images of Fig. 3A–C, yellow squares (sagittal) and lines (axial) are drawn that show the location and orientation, relative to the extrudate volume, where CLSM images were acquired. For visualisation, this indicator is enlarged ten times. The CLSM image of SPC extrudate at pH 5.0 shows a fibrillar protein structure. Correspondingly, the WOP(CLSM) map of this protein network shows regions with relatively high order, namely around 0.6 ± 0.2. Similar observations are done for the sample at pH 7.0. At pH 7.8, no clearly oriented protein network is observed at the 10^−1^ μm-scale and WOP(CLSM) values are low due to the absence of an aligned structure, which is expected for the disordered fibrillar network due to electrostatic inter-fibril repulsion at pH ≫ pI as shown by SANS and zeta-potential results in Kuijpers et al. (2025).

**Fig. 3:**
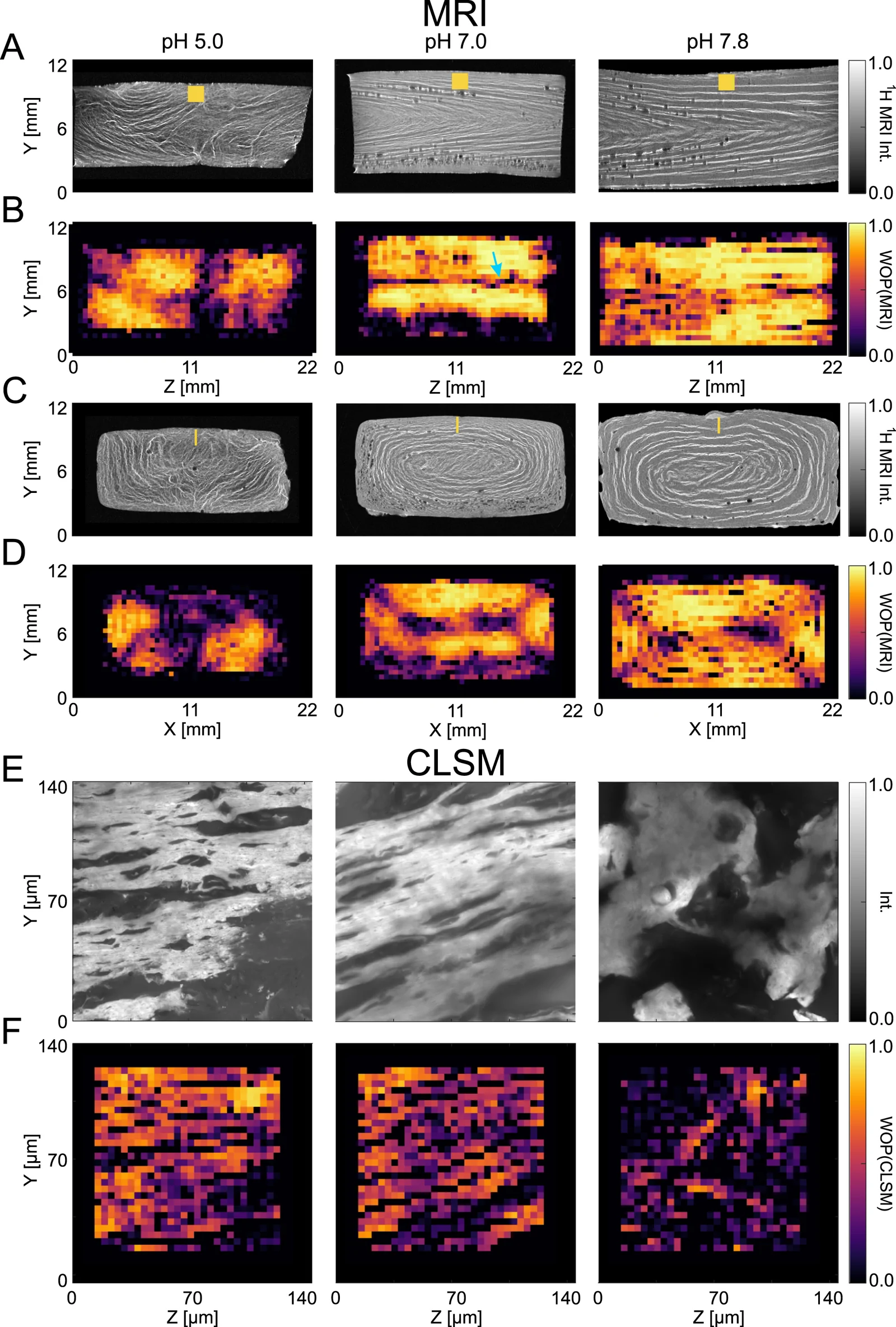
Imaging results for SPC extrudates at pH 5.0 (left), pH 7.0 (middle) or pH 7.8 (right): sagittal (A) or axial (C) MRI $T_{1}$- and $T_{2}$-weighted images, and CLSM images (E). Below each image, the corresponding sagittal and axial WOP(MRI) or WOP(CLSM) maps are shown (B, D, F). The relative sampling location of CLSM images is denoted by a yellow square (A) or by a yellow line (C) in the MRI images and is 10× enlarged for visualisation purposes. The cyan arrow highlights the region where two different orientations of phase-separated lamellae meet and WOP(MRI) values are lower.

### 3.3 Assessment of 10^−1^μm-scale anisotropic structure by DT-MRI

In the previous section, CLSM and MRI provided quantitative measures for anisotropic structure, respectively, at the 10^−1^ and 10^2^ μm length scales. In order to approach the 10^−1^ μm length scale with MRI, we measured the self-diffusive behaviour of water to probe structural anisotropy within the WR and PR lamellae. DT-MRI measurements enable per-voxel determination of a water self-diffusion tensor. In turn, this enables mapping of the mean diffusivity ($D_{av}$), the Fractional Anisotropy (FA) and the Principal Diffusion Direction (PDD) (see Fig. 4). The self-diffusion MRI map in Fig. 4A, referring to the SPC extrudate at pH 7.0, shows two distinct water regions with faster or slower self-diffusion, corresponding to the PR and WR phases seen in the $T_{2}$-weighted MRI data (Fig. 3A–C). The respective self-diffusion coefficients are $\left(9.4\pm 1.4\right)\cdot 10^{-10}$ m^2^/s and $\left(13\pm 1\right)\cdot 10^{-10}$ m^2^/s. Correspondingly, Fig. 4B shows two distinct populations with FA values of either (0.3 ± 0.1) or (0.2 ± 0.1). Distributions of both diffusion and FA maps are shown in Fig. S2.4 in the SI. Statistical analysis of these distributions revealed p<0.01. As shown by the CLSM data in Fig. 3E–F, the PR phase is an aligned protein network and, thus, it exhibits higher diffusional anisotropy compared to the WR phase. Using the Einstein equation one can estimate that for Δ in the range of 10–150 ms, self-diffusion of water is probed at a length scale of 9–33μm, which exceeds the 10^−1^ μm diameter of the aligned protein fibrils in the PR phase by several orders of magnitude. Hence we attribute the higher FA values for the PR phase to the presence of these aligned protein fibrils (See Fig. S1.2 in SI). Nevertheless, the FA values found in the WR phase indicate that the water self-diffusion is not fully isotropic in these regions. The deviation from fully isotropic diffusion may arise from non-negligible partial-volume effects for a slice thickness of 1 mm. Additionally, previous work has shown that, at pH ≫ pI, protein fibrils form a less aligned network due to inter-fibril electrostatic repulsion (Kuijpers et al., 2025). The SPC sample shifted to pH 5.0 shows water self-diffusion coefficients, and FA values similar to those for the sample at pH 7.0, which is in line with the fibrillar protein network observed by CLSM in Fig. 3E.

To acquire information about the orientation of the diffusion anisotropy, PDD(DT) maps are utilised to colour-code the orientation of the eigenvectors associated with each tensor eigenvalue. The PDD(DT) maps of three pH-shifted SPC extrudates are shown in Fig. 4C–E, for the primary (C), secondary (D) and tertiary (E) eigenvalues, respectively indicating the fastest, intermediate and slowest self-diffusion. To mitigate the effect of noise in the PDD maps, DT-MRI data is often pre-processed using denoising algorithms (Olesen et al., 2023). In this work, such denoising step yielded smoothed PDD maps at the expense of the resolution between WR and PR features in the FA maps. Thus, to retain such imaging details, smoothing of the PDD maps was performed solely by re-sorting the tensor eigenvalues using the ROI-method established in Basser and Pajevic (2000) outlined in Section 2.4.3 and Eq. (3). At pH 7.0, in the primary PDD(DT) map (Fig. 4C), *i.e.* the direction of the fastest self-diffusion, appears to be the Z-axis (regions in blue), which is parallel to the shear direction in the extruder. Concomitantly, in panel D and E, this colour is nearly absent. The secondary PDD(DT) map (Fig. 4D) unveils two separated populations with distinct self-diffusion direction along the cross-section of the cooling die. These regions are oriented along the Y-axis (regions in green) or along the X-axis (regions in red) in Fig. 4D and appear to be parallel to the lamellar phase-separated structure. In Fig. 4E, the orientations are inverted with respect to Fig. 4D. From this, it follows that the slowest diffusion direction in Fig. 4E is observed perpendicular to the lamellar phase-separated structures. These observations indicate that, inside the PR phase, an anisotropic phase-separated structure is present at a length scale below 20μm. Likely these are the 10^−1^ μm-scale protein fibrils observed in Fig. 3E that arrange in lamellae. These 10^−1^ μm-scale lamellae are oriented along the extruder flow direction and shear direction of the HME screws. Similar anisotropic diffusion features are observed for the sample shifted to pH 7.8, but a larger contribution of green (Y) and red (X) is visible in the primary PDD(DT) map (Fig. 4C), and vice versa, a larger contribution of blue (Z) is visible in the secondary PDD(DT) map (Fig. 4D). The distribution of colours remains unchanged in the tertiary PDD(DT) map (Fig. 4E) compared to that of the pH 7.0 sample. Together with the roughly 25% lower FA values observed in (Fig. 4B), this indicates that the tensor in these voxels is closer to a disk-like shape where the eigenvalues of the primary and secondary diffusion directions are similar in magnitude. In the PDD(DT) map of the sample shifted to pH 5.0, clear regions with a primary diffusion direction other than the extruder flow direction can be observed. Additionally, in the secondary as well as in the tertiary PDD(DT) map, non-uniformly coloured regions are observed. These observations are in line with the more disordered lamellar structure observed in Fig. 3C. This shows that, similarly to pH 7.0 and pH 7.8, the secondary diffusion direction is generally parallel to the lamellar phase separation, regardless of the direction of these lamellae.

**Fig. 4:**
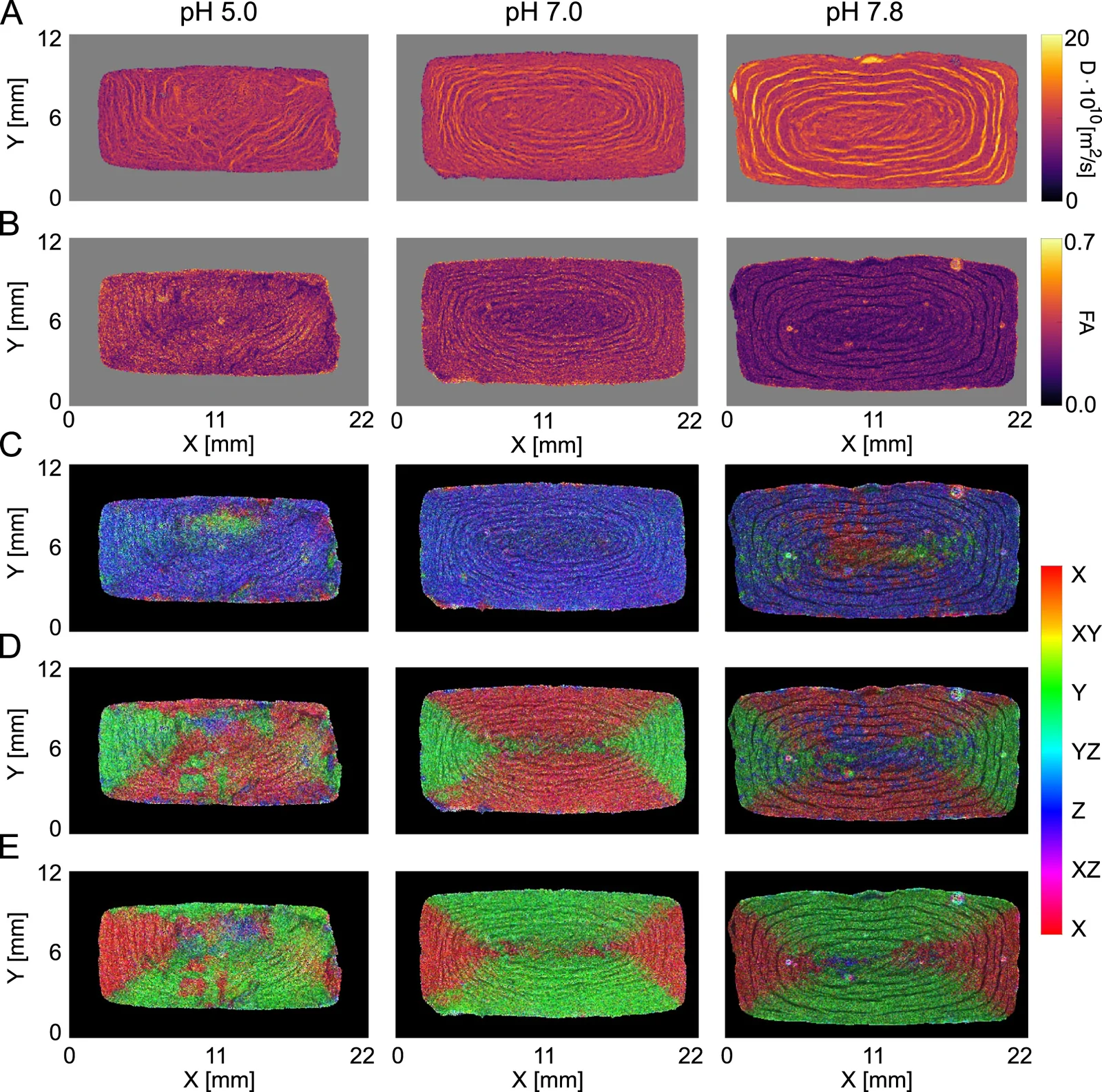
DT-MRI axial images of SPC extrudate samples at pH 5.0 (left), pH 7.0 (middle) or pH 7.8 (right). The maps in panels from top to bottom display: (A) the water self-diffusion coefficient, D (Eq. (1)), (B) the Fractional Anisotropy, FA (Eq. (2)), and the Principal Diffusion Direction (PDD) for either the primary (C), secondary (D) or the tertiary (E) tensor eigenvalues. The latter three images, respectively, correspond to the directions where self-diffusion is fastest, intermediate or slowest and the colour intensities are weighted by the corresponding FA value. These diffusion tensor eigenvalues are sorted by the ROI-based method established in Basser and Pajevic (2000), and the respective eigenvector directions are colour-coded for either X (red), Y (green) or Z (blue) direction.

**Fig. 5:**
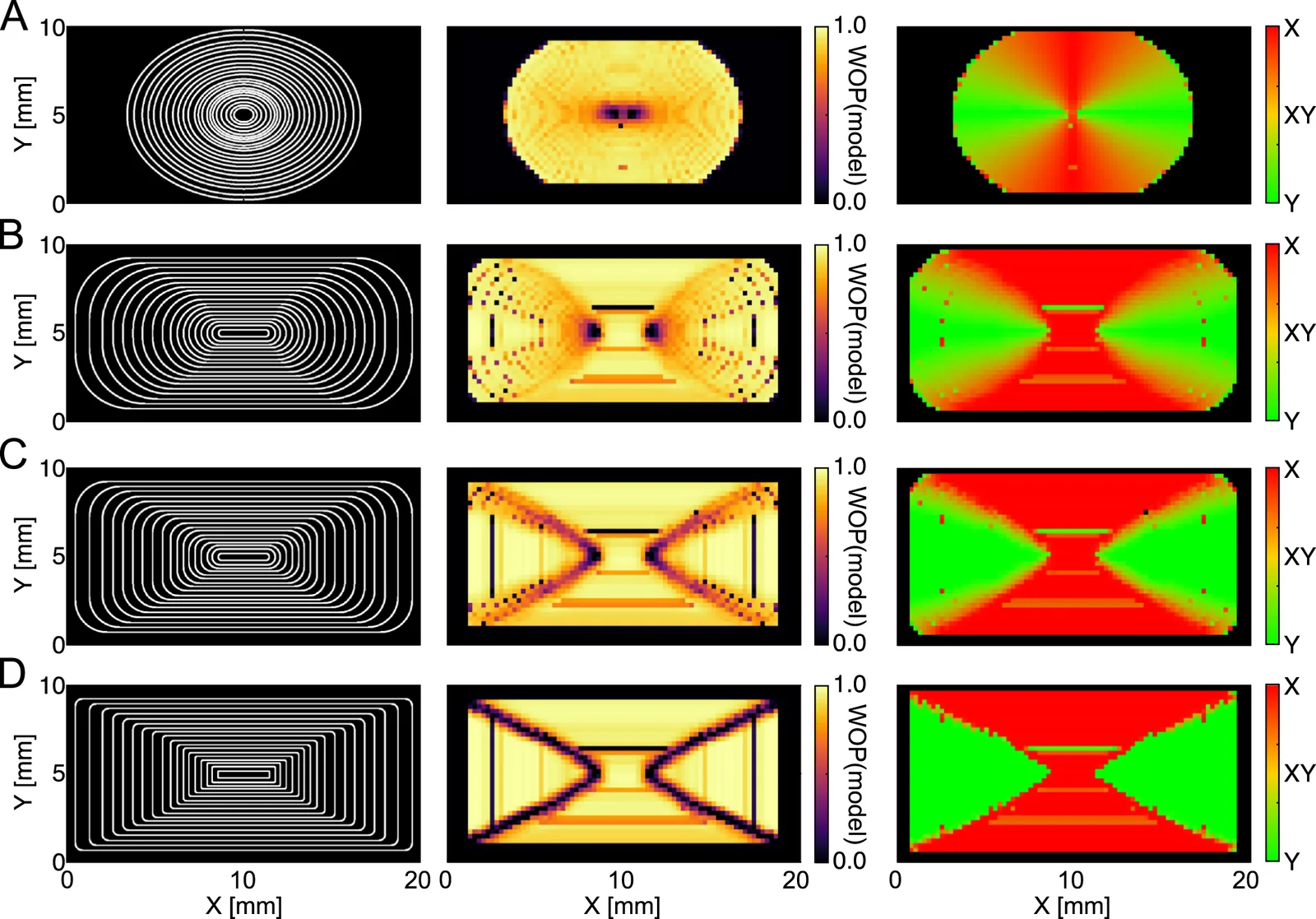
Synthetic axial images (left), mimicking the lamellar phase separation in SPC extrudates as seen by axial MRI data Fig. 3C, obtained for cross-sectional cooling die geometries with increasingly sharper corners ranging from elliptical (top) to rectangular (bottom) shaped. The synthetic MRI data, generated as described in Section 2.7, was further analysed by the RFT algorithm to create a vector field (data not shown). This vector field was used to calculate the corresponding WOP(model) (middle) and PDD(model) (right) maps.

### 3.4 The effect of cooling die geometry on lamellar phase-separation

In order to investigate the influence of the cross-sectional cooling die geometry on the morphology of the phase-separated lamellar structure of SPC extrudates, synthetic axial MRI images were generated, as described in Section 2.7. In Fig. 5A, the simulated axial image (left) for a cooling die with an ellipse-like cross-sectional geometry, along with its WOP(model) (middle) and PDD(model) (right) maps, are shown. The latter maps (Fig. 5), respectively simulating the 10^2^ (Fig. 3D) or 10^−1^ μm length scale (Fig. 4C), were calculated using the same RFT algorithm adopted for the WOP(MRI) maps (see section Section 2.6). In Fig. 5A, the orientation and curvature of the lamellar phase-separated structure evolves smoothly along the XY plane as expected, without sharp corner regions. Correspondingly, the WOP(model) values for this data are (∼0.93 ± 0.02), determined as outlined in Fig S2.6 in the SI, and reflect a highly ordered, homogeneous, ellipse-like lamellar structure (Fig. 5A). These findings are consistent with DT-MRI and WOP(MRI) maps acquired for a pilot-scale SPC extrudate at pH 7.0, produced as described in Garina et al. (2026) using a cooling die with fully rounded edges (See SI Fig S2.5). The latter data revealed a uniform WOP(MRI) distribution even across the rounded-edge region (See SI Fig S2.5 A-B), in analogy to the WOP(model) results in Fig. 5A. In the corresponding PDD(model) maps (Fig. 5A, right), simulating the 10^−1^ μm-scale, the vector angle orientation is seen to gradually evolve between X (red), at the top and bottom, and Y (green), at the left and right sides of the map, with combinations of X-Y directions being displayed in a gradient (orange).

In Fig. 5B–D, the synthetic axial images, WOP(model) and PDD(model) maps for cross-sectional cooling-die geometries with increasingly sharper rectangular profiles are shown. These geometries display progressively more abrupt changes at the corner edges in the axial plane, in the spatial orientation of the lamellar phase-separated structure. The WOP(model) map in Fig. 5B shows a value of (0.96 ± 0.02) in the region where structure is oriented horizontally (see SI Fig S2.6). In the corner regions, the WOP(model) value decreases to (0.9 ± 0.1) with the more disordered corner region having a width of 3.3 mm (see SI Fig S2.6). In panel C, WOP(model) values decrease from (0.97 ± 0.01) in the horizontally ordered region to (0.6 ± 0.2) in the more disordered corner region. The width of this disordered region decreased to 1.9 mm. By creating a sharper rectangular profile, with a disordered corner region of 1.1 mm (Fig. 5D), WOP(model) values decrease from (0.97 ± 0.01) in the horizontally ordered region to (0.3 ± 0.2) in the disordered corner regions. The WOP(MRI) map in Fig. 3D, of the sample at pH 7.0, shows a decrease in WOP values similar to those found in the simulations shown in Fig. 5D, which is simulating the geometry of the cooling die used in this work. Associated WOP values are (0.88 ± 0.07) and (0.5 ± 0.2) respectively for the ordered middle region and disordered corner region. The width of the disordered corner region is approximately 1.5 mm. When considering the PDD(model) maps for the rectangle-like shape with rounded corners (Fig. 5B, right), the spatial variation of the vector angle appears similarly gradual to the ellipse-like shape shown in Fig. 5A. When the rounded corners become sharper, the corresponding X-Y transition region in the PDD(model) map rapidly decreases in thickness. The secondary diffusion direction observed for the sample shifted to pH 7.0 (Fig. 4D, right), shows an angle distribution similar to the simulated PDD(model) map in Fig. 5C. We notice that, for the pilot-scale sample at pH 7.0, an angle distribution more similar to Fig. 5B (right) is observed (see SI Fig S2.5E).

**Fig. 6:**
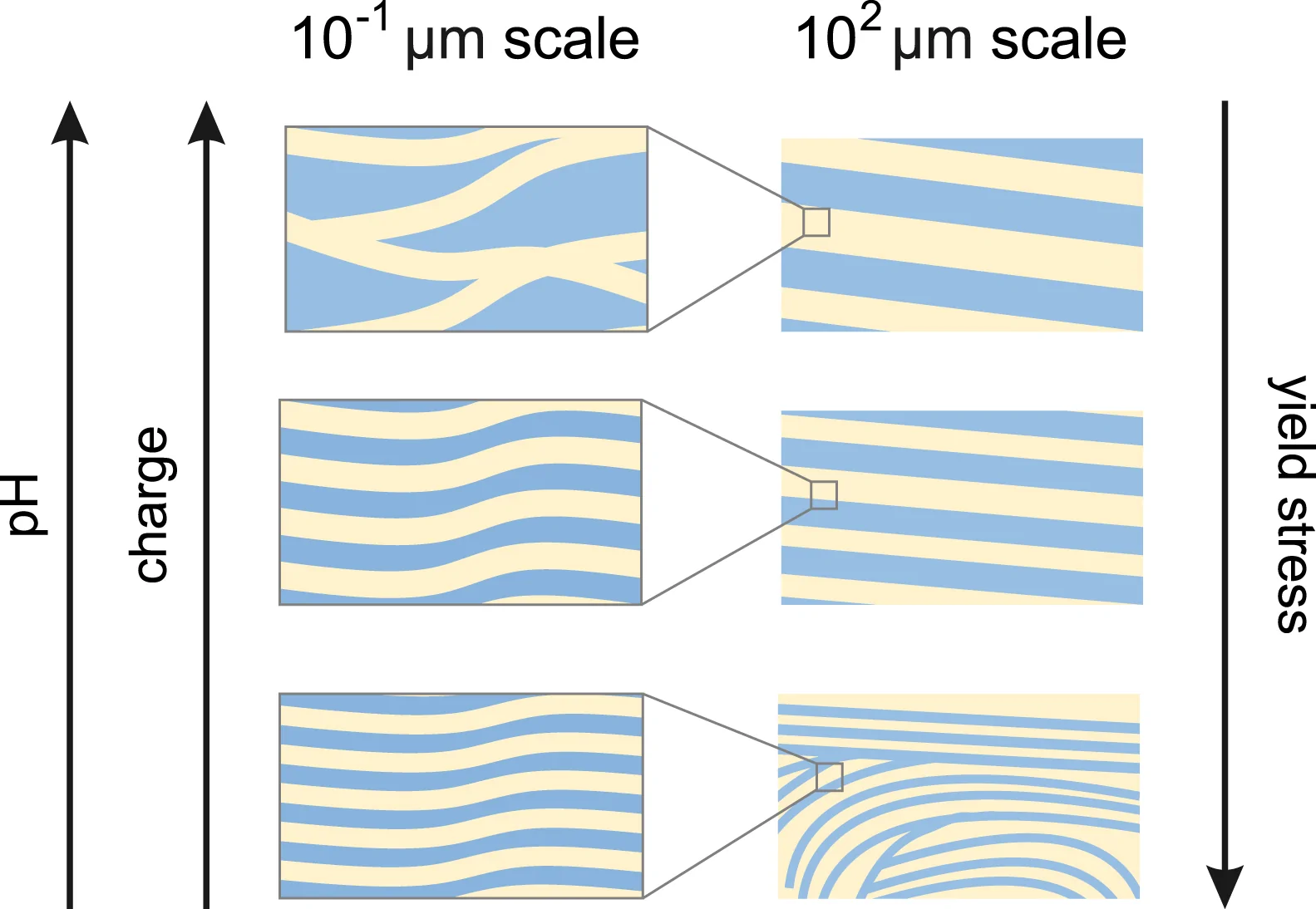
Schematic representation of anisotropic structure formation during extrusion at the 10^−1^μm-scale, likely occurring already in the barrel section, and at 10^2^μm-scale, predominantly occurring inside the cooling die. The blue and yellow colours respectively represent water- and protein-rich domains. At alkaline pH, alignment of protein fibrils at the 10^−1^μm-scale is lacking, due to high inter-fibril electrostatic charge repulsion, resulting in low yield stress of the protein melt and lamellar structure formation at the 10^2^μm-scale (top). When a balance is struck between fibrillar alignment and their electrostatic repulsion at the 10^−1^μm-scale, an aligned fibrillar protein network forms that still exhibits laminar flow behaviour in the cooling die (middle). At acidic pH, closer to the pI, the aligned fibrillar protein network at the 10^−1^μm-scale has sufficiently increased the yield stress of the protein melt to hamper the laminar flow behaviour inside the cooling die, resulting in a disordered phase-separated structure at the 10^2^μm-scale (bottom).

### 3.5 Structural hierarchy at 10^−1^–10^2^μm length scale in SPC extrudates

The CLSM images in Fig. 3E–F and DT-MRI FA data in Fig. 4B show that an aligned fibrillar protein network is observed, at the 10^−1^ μm-scale, for the SPC extrudates at pH 5.0 and 7.0. This indicates that pH-shifting down to values closer to the pI, near pH 5.0, leads to an isotropic particulate protein network (Kuijpers et al., 2025). Also at the 10^2^ μm-scale, a lack of an aligned lamellar structure is observed by MRI images and WOP(MRI) maps in Fig. 3A–D at pH 5.0 as schematically represented in the bottom row of Fig. 6. The structuring at this length scale has been shown to primarily occur in the cooling die of the extruder (Garina et al., 2026). The pH of the protein melt affects its rheological properties and, thus, flow behaviour occurring inside the cooling die. At pH 5.0, the yield stress of the protein melt is higher compared to that at pH 7.0 and 7.8 (Gobes et al., 2025a). The stress applied to the protein melt at pH 5.0 is likely not sufficiently high to create the sheared laminar flow through the cooling die that results in the parallel lamellae seen in Fig. 3A at pH 7.0 and 7.8. However, at the 10^−1^ μm-scale, the phase-separated structure observed by PDD(DT) maps closely follows the orientation of the phase-separated lamellae observed at the 10^2^ μm-scale. Based on these results, we hypothesise that structure formation at the 10^−1^ μm-scale precedes that of phase-separated structuring in the cooling die. This is supported by the work of Garina et al. (2026), where it was shown that formation and alignment of small-scale fibrils already occurs in the barrel section and transition zone, between the extruder barrel and cooling die. Likely, the corresponding formation of a particulate network as a result of low charge shielding among the protein fibrils (Kuijpers et al., 2025), contributes to the high yield stress of the protein melt and the hampering of formation of macroscopic aligned lamellar structure during passage through the cooling die. This indicates that an optimum pH exists at which (i) fibrillar network alignment occurs at the 10^−1^ μm-scale and (ii) the yield stress of the protein melt is sufficiently low to drive lamellar phase separation, at 10^2^ μm-scale, during flow through the cooling die (Kuijpers et al., 2025, Gobes et al., 2025a, Garina et al., 2026). These insights have been schematically summarised in Fig. 6.

**Fig. 7:**
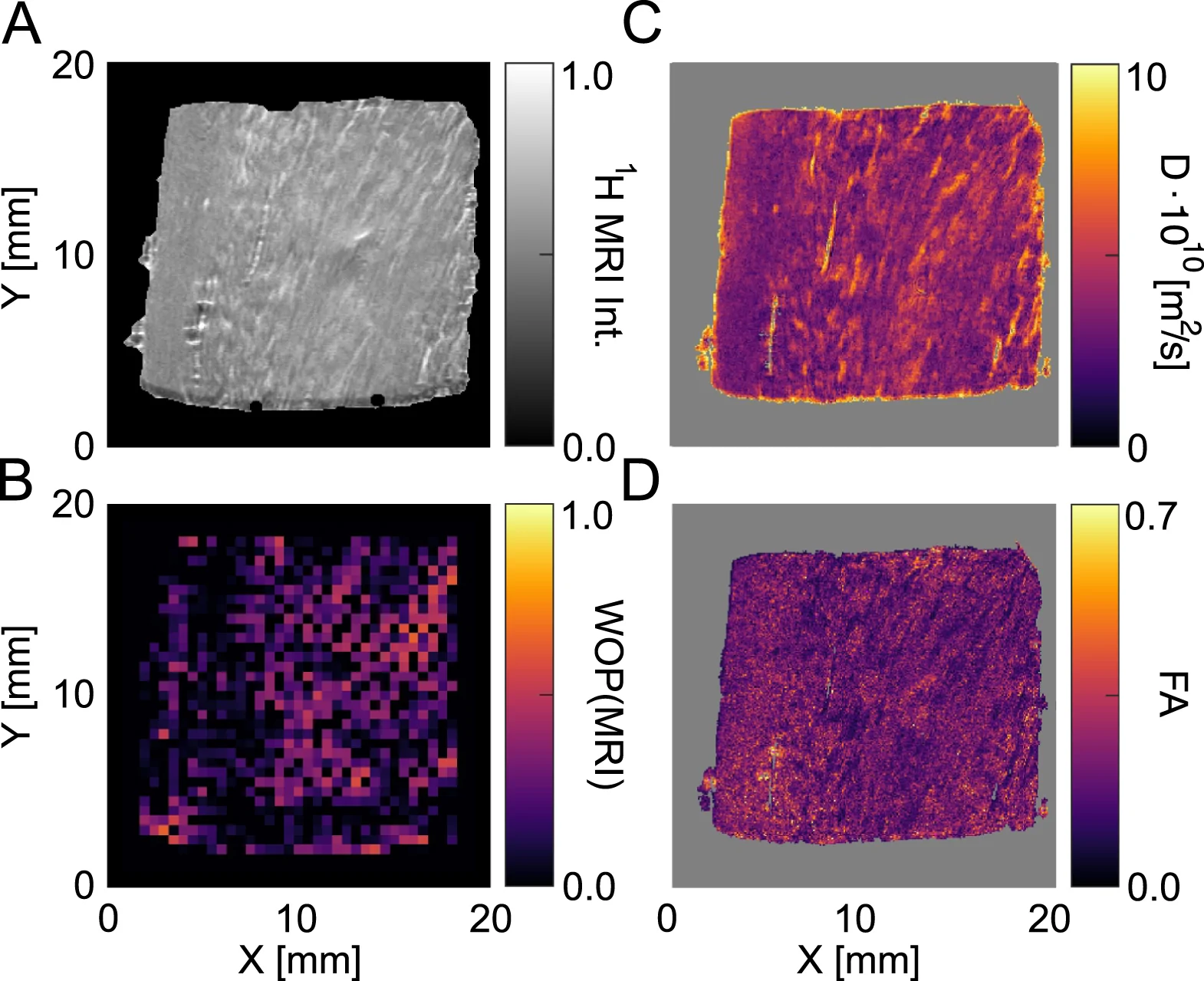
MRI results for commercial high-moisture bovine mozzarella cheese: $T_{1}$- and $T_{2}$-weighted RARE MRI (A) and respective WOP(MRI) (B) results, along with maps of apparent diffusion coefficient (C) and FA (D), obtained from DT-MRI STE measurements and diffusion tensor calculations. The background signal was removed from the image by masking of the pixels outside the sample area.

### 3.6 MRI and DT-MRI assessment of hierarchical structure in mozzarella

In this work, we used $T_{2}$-weighted MRI and DT-MRI approaches to investigate the hierarchical structure of commercial high-moisture mozzarella, to show the applicability of our method to another structured protein matrix. Mozzarella is industrially produced through the heating, between 60 – 80 °C, and stretching of full fat or skim cheese curd (Tremonte et al., 2024, Yu and Gunasekaran, 2005). The stretching of the heated curd is what determines the parallel alignment of casein fibrils and imparts structure. In Fig. 7, MRI and DT-MRI results of a commercial high-moisture bovine mozzarella (∼64 w%), are shown. In Fig. 7A, an phase-separated structure is distinguishable with a homogeneous alignment parallel to the left sample edge, which is the exterior of the sample relative to the uncut mozzarella product. In the WOP(MRI) map Fig. 7B, this region is marked as having high anisotropic order at the 10^2^ μm length scale. Similar results are observed in microscopy images of high-moisture (∼56 wt%) bovine-mozzarella cheese shown in Nguyen et al. (2017). However, an aligned anisotropic structure is not observed at the lower length scale probed by DT-MRI and shown in the FA map in Fig. 7D. The absence of anisotropic alignment at such lower length scales is confirmed by SANS measurements, which showed isotropic scattering profiles at length scales below 90 nm (Fig S3.1 in SI). Scattering profiles remained isotropic after contrast enhancement using D_2_O (Garina et al., 2024). Recent SANS work on caseinate dispersions that have undergone strong thermo-mechanical treatment shows that a hierarchical anisotropic structure can form (Tian et al., 2020). Likely, the thermo-mechanical processing applied during the production of mozzarella cheese is not sufficient to induce such structure formation at the length-scales probed by SANS. These results indicate that nm-scale structural alignment is required to observe 10^−1^ μm-scale aligned structures by DT-MRI, such as is the case for SPC extrudates where nm-scale protein fibrillar alignment is clearly observed by SANS measurements (Kuijpers et al., 2025, Garina et al., 2026).

**Fig. 8:**
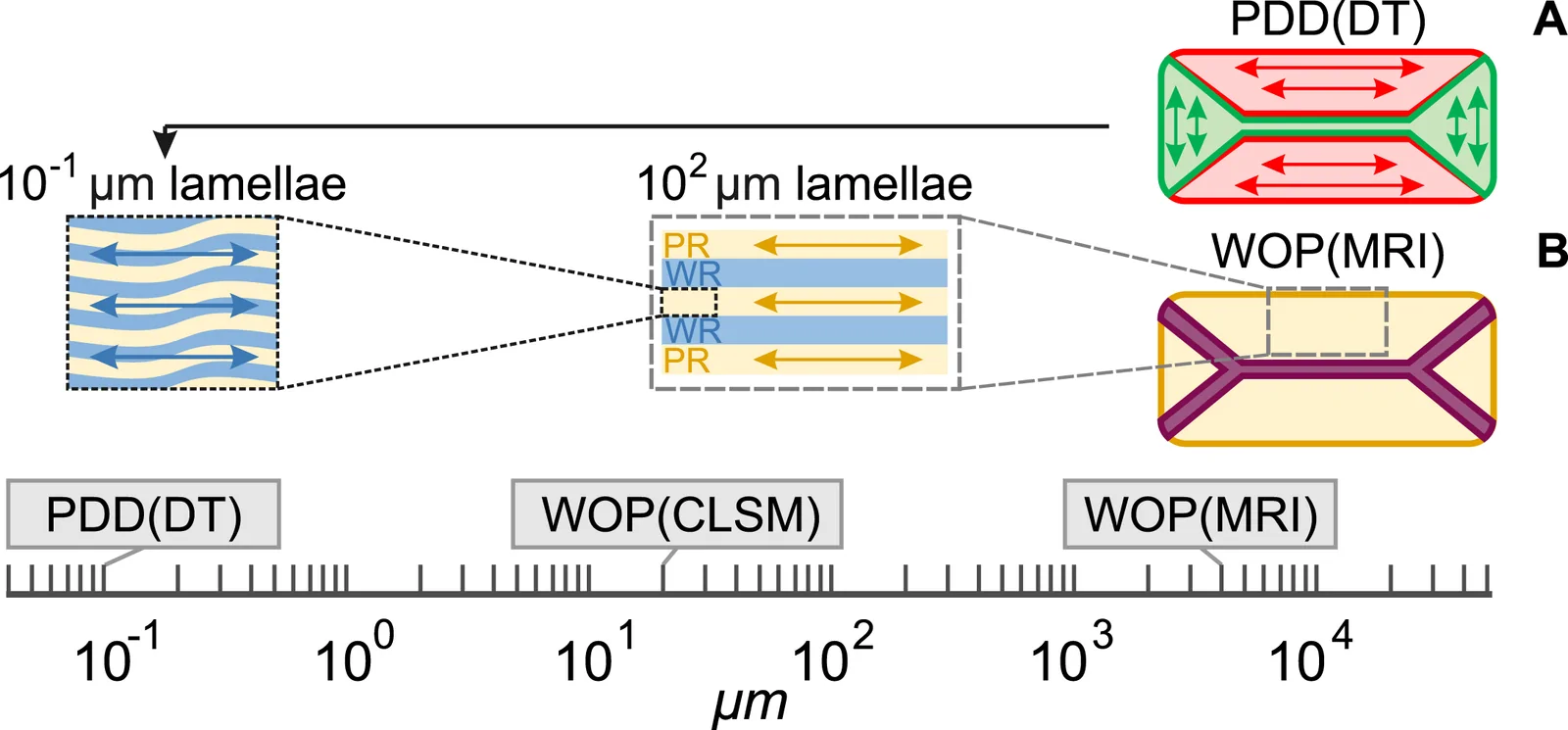
Schematic diagram of the hierarchy in anisotropic structure of SPC extrudates. (A) DT-MRI PDD(DT) maps where the water self-diffusion direction, hindered by the protein fibrils seen in CLSM (Fig. 3E) at the $10^{-1}\;\mu m$-scale, is colour-coded for X (red) and Y (green) as shown in Fig. 4D; (B) WOP(MRI) maps of $10^{2}\;\mu m$-scale lamellar alignment (Fig. 3D), where high alignment (yellow) is observed along the sample edges and low alignment (purple) where two or more alignment directions meet. Here, alignment is averaged over a $∼4\cdot 10^{3}\;\mu m$-scale area; in the WOP(CLSM) maps (Fig. 3F) protein fibril alignment is averaged over a $∼2\cdot 10^{1}\;\mu m$-scale area.

## 4 Conclusions

In this work, we investigated the hierarchical structure of pH-shifted SPC extrudates on a wide range of length scales, from 10^−1^ μm (Fig. 8A) to 10^2^ μm (Fig. 8B). To cover such wide range of structural length scales, we exploited an array of techniques, including CEST MRI, $T_{2}$-weighted and DT-MRI mapping, as well as advanced image analysis and simulation methods.

In SPC extrudates, RFT analysis of $T_{2}$-weighted MRI images unveiled aligned PR and WR lamellae at the 10^2^ μm length scale (Fig. 8B) at pH 7.0 and 7.8, but a more disordered lamellar structure at pH 5.0. CEST MRI showed the presence of mobile proteins in the WR lamellae. Based on DT-MRI results, these dissolved proteins do not contribute to the formation of anisotropic structures in the WR lamellae. DT-MRI showed that the PR lamellae had an underlying anisotropic structure at the 10^−1^ μm length scale, which can be semi-quantified by PDD maps (Fig. 8A). The presence of structural anisotropy at this small length scale was confirmed by CLSM, which showed alignment of protein fibrils at the 10^−1^ μm length scale at pH 5.0 and 7.0. The MRI-based RFT and PDD maps provided a quantitative measure of the impact of pH-shifting on hierarchical anisotropic structure formation in the cooling die and showed how fibrillar alignment at the 10^−1^ μm length scale may impact lamellar phase-separation at the 10^2^ μm length scale. The detailed MRI features observed in the RFT and PDD maps of SPC extrudates could be explained by simulating the effect of the cross-sectional cooling die geometry.

We note that the observed hierarchical character of structural anisotropy is specific to SPC extrudates. In a different anisotropic protein-based material, here mozzarella, we observed order only at the 10^2^ μm length scale. Due to the lack of order at lower length scales, DT-MRI showed no evidence of diffusional anisotropy.

We conclude that high-resolution MRI is a versatile measurement technique suitable to characterise hierarchical anisotropic structures in extruded protein-based food materials. In particular we envisage a role for DT-MRI and MRI for unravelling the interplay 10^−1^ μm-scale anisotropic structuring of protein and rheological yield stress behaviour impacting on phase-separated structure formation at the 10^2^ μm length scale.

## CRediT authorship contribution statement

**Sam A. Kuijpers:** Conceptualization, Data curation, Formal analysis, Investigation, Methodology, Visualization, Software, Writing – original draft. **Martijn I. Gobes:** Data curation, Investigation, Software. **Morwarid Mayar:** Data curation, Investigation, Methodology. **Frank J. Vergeldt:** Methodology, Software, Data curation, Writing – review & editing. **Johannes Hohlbein:** Writing – review & editing. **Thom Huppertz:** Writing – review & editing. **John P.M. van Duynhoven:** Conceptualization, Funding acquisition, Resources, Supervision, Writing – review & editing. **Camilla Terenzi:** Conceptualization, Funding acquisition, Resources, Supervision, Writing – review & editing, Project administration.

## Declaration of competing interest

The authors declare the following financial interests/personal relationships which may be considered as potential competing interests: John van Duynhoven reports a relationship with Unilever Innovation Centre Wageningen BV that includes: employment. Thom Huppertz reports a relationship with Koninklijke FrieslandCampina NV Innovation Centre Wageningen that includes: employment. If there are other authors, they declare that they have no known competing financial interests or personal relationships that could have appeared to influence the work reported in this paper.

## Data Availability

Data can be made available on request.

## Appendix A Supplementary data

MMC S1 . MMC S1
