# Supplementary material for: Underpinning hierarchical anisotropic structure formation in soy protein high-moisture extrudates by Magnetic Resonance Imaging
Source: Curr Res Food Sci. 2026 Sep 26;13:101575. doi: 10.1016/j.crfs.2026.101575 (PMC13651476; doi:10.1016/j.crfs.2026.101575)
Supplement: Supplementary file 1 [file mmc1.pdf]

Supplementary Information for:

Underpinning Hierarchical Anisotropic Structure Formation in Soy Protein High-Moisture Extrudates by Magnetic Resonance Imaging

Sam A. Kuijpers[a], Martijn I. Gobes[a], Morwarid Mayar[a], Frank J. Vergeldt[a], Johannes Hohlbein[a,b], Thom Huppertz[c], John P.M. van Duynhoven[a,d,*] and Camilla Terenzi[a,*]

[a] *Laboratory of Biophysics, Wageningen University & Research, Stippeneng 4, 6708 WE Wageningen, The Netherlands*
[b] *Microscopy Research Facility, Wageningen University & Research, Stippeneng 4, 6708 WE Wageningen The Netherlands*
[c] *FrieslandCampina, Stationsplein 4, 3818 LE Amersfoort, The Netherlands*
[d] *Unilever Global Foods Innovation Centre, Bronland 14, 6708 WH Wageningen, The Netherlands*

Highlights:

- SPC extrudates display hierarchical anisotropic structure at the 10^{-1} – 10^{2} μm length scale.
- T_2-weighted spin echo MRI quantifies anisotropy at the 10^{2} μm-scale.
- Diffusion Tensor MRI probes anisotropy at the 10^{-1} μm-scale.

Table of Contents

S1: Image acquisition 3

S1.1: Sample preparation 3

S1.2: Effect of diffusion mixing time on tensor calculations 4

S1.3: Effect of signal-to-noise ratio on tensor calculations 5

S1.4: Diffusion reference image acquisition 6

S1.5: Effect of number of diffusion directions on tensor calculations 7

S1.6 ROI-based Eigenvalue sorting 8

S2: Additional CEST and DT-MRI results 9

S2.1: CEST of SPI and methylcellulose dispersion 9

S2.2: CEST of soaked fresh SPC extrudate 10

S2.3: WOP histograms and statistical analysis 11

S2.4: Histograms of DT-MRI diffusion and FA maps 12

S2.5: MRI and DT-MRI of pilot-scale SPC extrudate 13

S2.6: Mean WOP areas of synthetic cross-sectional cooling die data 14

S3 Nano-scale structure assessment of mozzarella cheese 15

S3.1 SANS data of mozzarella cheese 15

References: 16

S1: Image acquisition

S1.1: Sample preparation

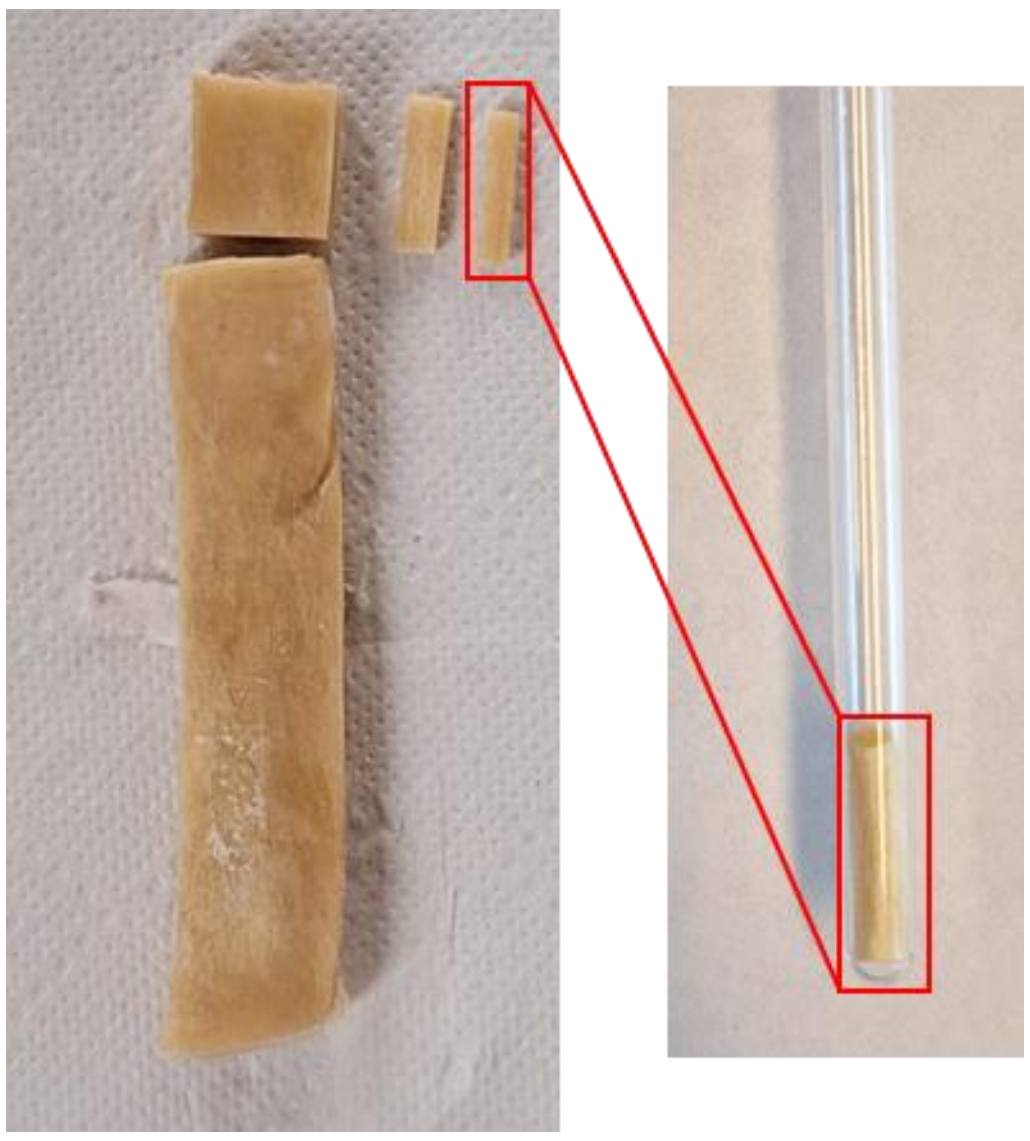

Figure S1.1. Photograph of the extrudate sample used for the CEST MRI measurements.

S1.2: Effect of diffusion mixing time on tensor calculations

In order to observe the diffusional anisotropy of water inside an SPC extrudate, hindrance of water diffusion by aligned protein fibrils should be effective. These protein fibrils have diameters in the 10^{-1} µm (Gobes *et al.*, 2025, *Food Struct.* 44, 100437). To ensure that water experiences hindered diffusion by these aligned protein fibrils, a diffusion mixing should be chosen such that mean square displacement of water exceed the 10^{-1} µm length scale by orders of magnitude. Using Einstein's formula for 3D isotropic diffusion a diffusion radius, $\langle r^2 \rangle$ of 9 µm – 33 µm can be expected for Δ values between 10 and 150 ms (Fig S1.2). This estimate is based on an average diffusion coefficient of water inside an SPC extrudate of $1.25 \cdot 10^{-9}$ m^2/s (Fig S1.2B). We note that the Einstein formula provides an upper limit for the diffusion radius since it is based on the assumption of isotropic diffusion in three dimensions. Nevertheless we can assume that for Δ values between 10 and 150 ms a length scale is probed that exceeds the fibril diameter by orders of magnitude.

Fig. S1.2A shows that the relative amount of physically inaccurate tensor elements, i.e. with negative eigenvalues, increases with increasing Δ. This occurs due to the decreased SNR resulting from the additional T_1-weighing at longer mixing times in an STE acquisition (See S1.3). Fig. S1.2B shows that, with increasing Δ, the mean diffusion coefficient shifts down. However, due to the longer mixing times required, the minimum effective b-value used for the A0-images increased from 120 s/mm^2 to 330 s/mm^2, which shifts the slope of the linear least-squares regression towards lower diffusion coefficient values. Additionally, a shift in the FA values towards 1 can be seen in Fig S1.2C similar to data shown in Fig S1.3C, due to a decrease in SNR.

Based on the results of Fig S1.2, a Δ of 50 ms was chosen, corresponding to a $\langle r^2 \rangle$ of roughly 20 µm, which is the largest mixing time that still results in less than 5% of voxels returning negative tensor eigenvalues and the diffusion coefficient does not notably change for the PR phase.

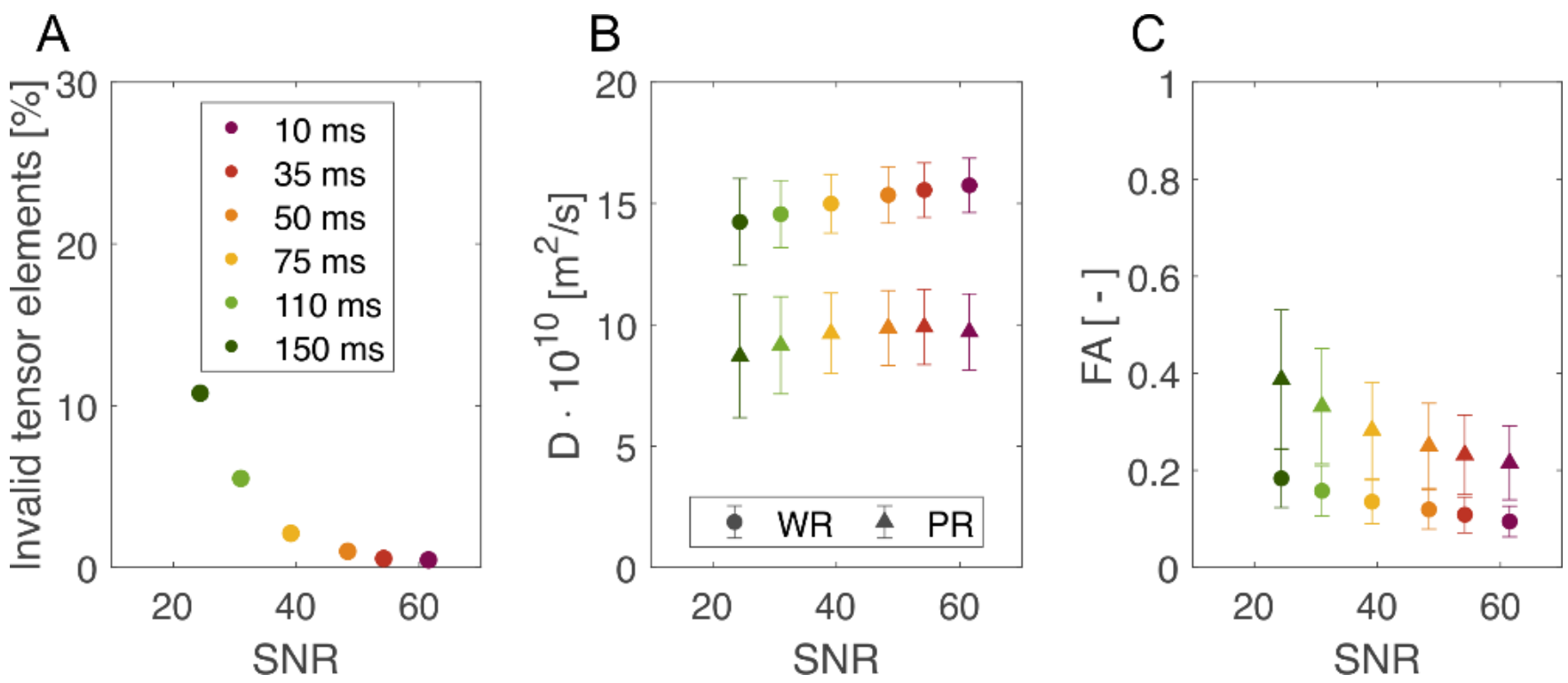

Fig. S1.2: *DT-MRI results acquired on a pH 7 SPC extrudate using an increasing mixing time for diffusion. Data was acquired using 6 manually set A0-images at b_{eff} = 120 s/mm^2 (b_{eff} = 250 s/mm^2 at Δ = 110 ms and b_{eff} = 330 s/mm^2 at Δ = 150 ms) and 32 images at b = 1000 s/mm^2. Acquisition parameters were as follows: STE with TE = 12 ms; TR = 1000 ms; voxel size = 78x78x1000 µm^3; δ = 2 ms and 4 averages were used. Images were masked for the background. (A) relative amount of non-physical tensor elements assessed by reviewing the number of voxels with one or more negative tensor elements. (B) Distribution of diffusion coefficients calculated from the trace of the diffusion tensors. (C) Distribution of fractional anisotropy values calculated per voxel from the tensor eigenvalues.*

S1.3: Effect of signal-to-noise ratio on tensor calculations

When either of the two datapoints, used by the linear regression model to calculate the diffusion value, has a significant enough contribution from noise, such that it cannot be distinguished from a measurement in a different gradient direction, the tensor calculation can become erroneous (Kingsley 2006b). Fig. S1.3 shows the results of an experiment where the noise was artificially increased by reducing the number of sampling averages per acquisition. One way to observe the effect of erroneous tensor calculations is to review how many tensors have one or more negative eigenvalues (Kingsley 2006b). Fig. S1.3A shows that as the SNR decreases, the relative amount of erroneous tensor calculations goes up. Fig S1.3B shows that only a minor effect on the diffusion coefficient is found for both the WR and PR phases. However, the effect is more noticeable on the FA calculation. A decrease in SNR is associated with an increase in FA and is not a physical property of the sample but the result of inaccurate tensor calculations. The data shows that the optimal SNR for this sample at the chosen resolution is between 4 and 6. Based on the data shown in Fig S1.3, the data in the main text figures was acquired using 8 averages due to the smaller voxel size of 52x52x1000 μm^3.

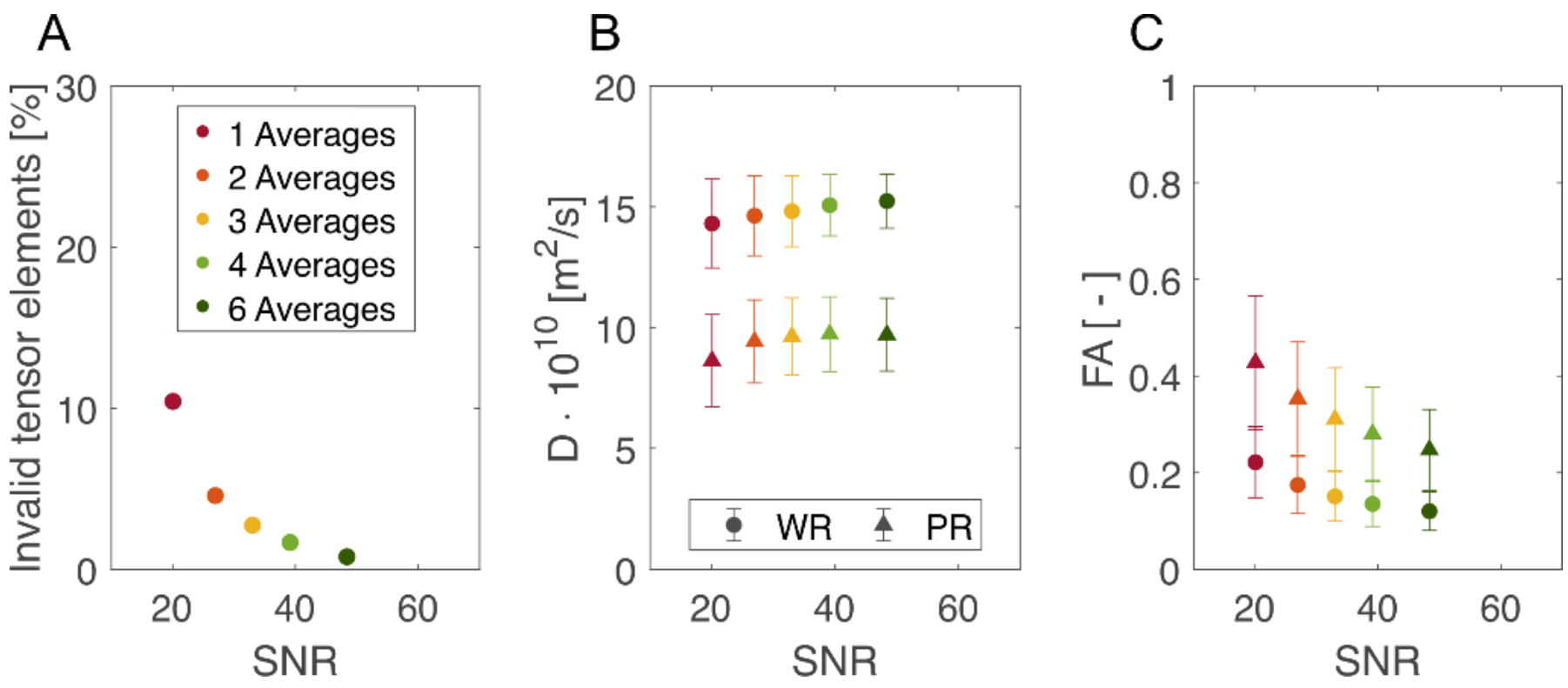

Fig. S1.3: *DT-MRI results acquired on a SPC extrudate at pH 7 using an increasing number of averages to shift the SNR of the acquisition. Data was acquired using 6 manually set A0-images at* b_{eff} *= 120 s/mm²* *and 32 images set at a b-value of 1000 s/mm². Acquisition parameters were as follows: STE with TE = 12 ms; TR = 1000 ms; voxel size = 78x78x1000* μm^3*;* δ *= 2 ms and* Δ *= 50 ms. Images were masked for the background. (A) relative amount of non-physical tensor elements assessed by reviewing the number of voxels with one or more negative tensor elements. (B) Distribution of diffusion coefficients calculated from the trace of the diffusion tensors. (C) Distribution of fractional anisotropy values calculated per voxel from the tensor eigenvalues.*

S1.4: Diffusion reference image acquisition

Due to a bug in the Paravision software version used in this work, the b-values are incorrectly calculated when using the stimulated-echo sequence, resulting in consistently underestimated diffusion coefficients of free water as shown by Fig S1.4. As a workaround, the A0-images were manually set as the first experimental shell with a minimum b_{eff} of 120 s/mm^2, resulting from the imaging and spoiler gradients.

Fig S1.4AC shows that the use of the built-in A0 images in Paravision leads to the underestimation of the diffusion coefficient which now becomes dependent on Δ. In a conventional DWI measurement using a multitude of b-values, this can be easily alleviated by omitting these values from the fitting as shown in Fig S1.4BD. However, for DT-MRI generally only two b-values are used, those of the A0-images close to 0 s/mm^2 and one between 800 and 2000 s/mm^2 depending on the system (Kingsley 2006abc). The data is typically fitted using a linear least squares algorithm and due to the limited b-values, this fit is largely dependent on the correct acquisition of the A0-images.

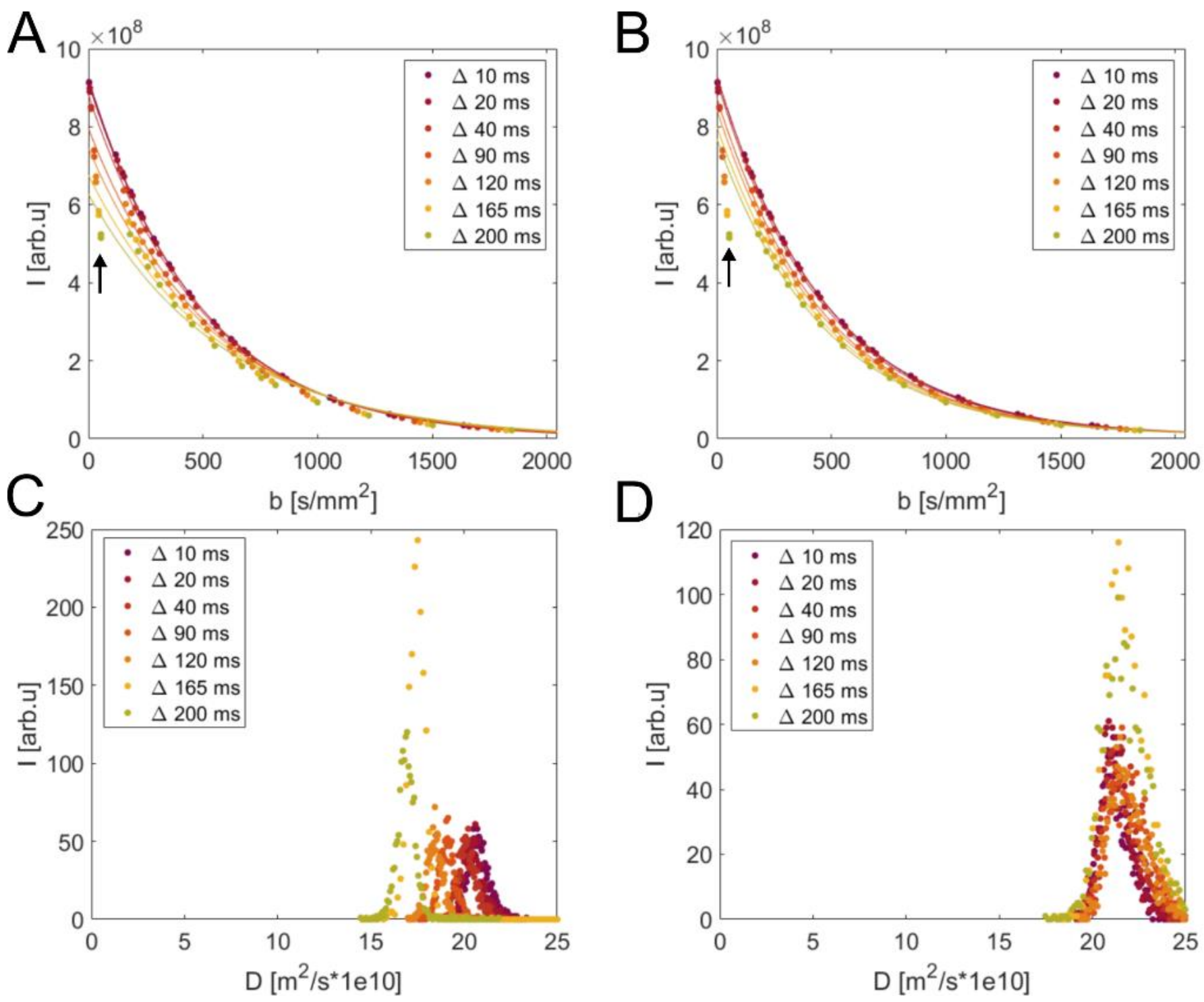

Fig. S1.4: *DWI results acquired on a 10 mm tube of demineralised water using different mixing times in a STE sequence. Two A0-images were acquired and 14 b-values were geometrically chosen between 110 s/mm^2 and 1600 s/mm^2. The resulting images were masked for the background and the mean intensity of the water was plotted. (A) DWI intensity results fitted using an exponential decay including the A0 image intensity. (B) DWI intensity results fitted using an exponential decay excluding the A0 images from fitting. The black arrow points to the A0 image intensities. (C) The distribution of diffusion values acquired from per voxel exponential fitting of the intensity decay data including the A0 image intensities. (D) The distribution of diffusion values acquired from per voxel exponential fitting of the intensity decay data excluding the A0 image intensities. The expected diffusion coefficient of water at the room temperature of 21°C is $2.07 \cdot 10^{-9}$ m^2/s.*

S1.5: Effect of number of diffusion directions on tensor calculations

A minimum of six diffusion directions along the six unique cartesian vectors (xx, yy, zz, xy, xz, yz) are required to calculate the diffusion tensor. However, if just one of these directions contains noisy data, the entire tensor calculation can become inaccurate (Kingsley 2006b). Therefore, it is advised to include the acquisition of additional diffusion encoding directions, as well as, spacing these directions not along the six unique cartesian vectors but instead on a hemisphere (Kingsley 2006b). The inclusion of more than six diffusion acquisitions will inherently decrease the sensitivity of the tensor calculation to noise. Additionally, by choosing uniformly distributed diffusion encoding directions, the anisotropy of hindered diffusion can be more accurately assessed, at the cost of longer measurement times. In the Fig. S1.5, different encoding schemes of increasing number of directions have been used to acquire a DT-MRI image of a pH 7 SPC extrudate. The imaging parameters were kept constant, thus, the SNR between acquisitions is constant. However, the amount of invalid tensor elements decreases as more imaging directions are added, showcasing the effect noise has on the tensor calculation. Between 32 and 48 directions, the number valid tensor elements and resulting FA calculations remained constant with increasing directions. In this work, 36 imaging directions are used.

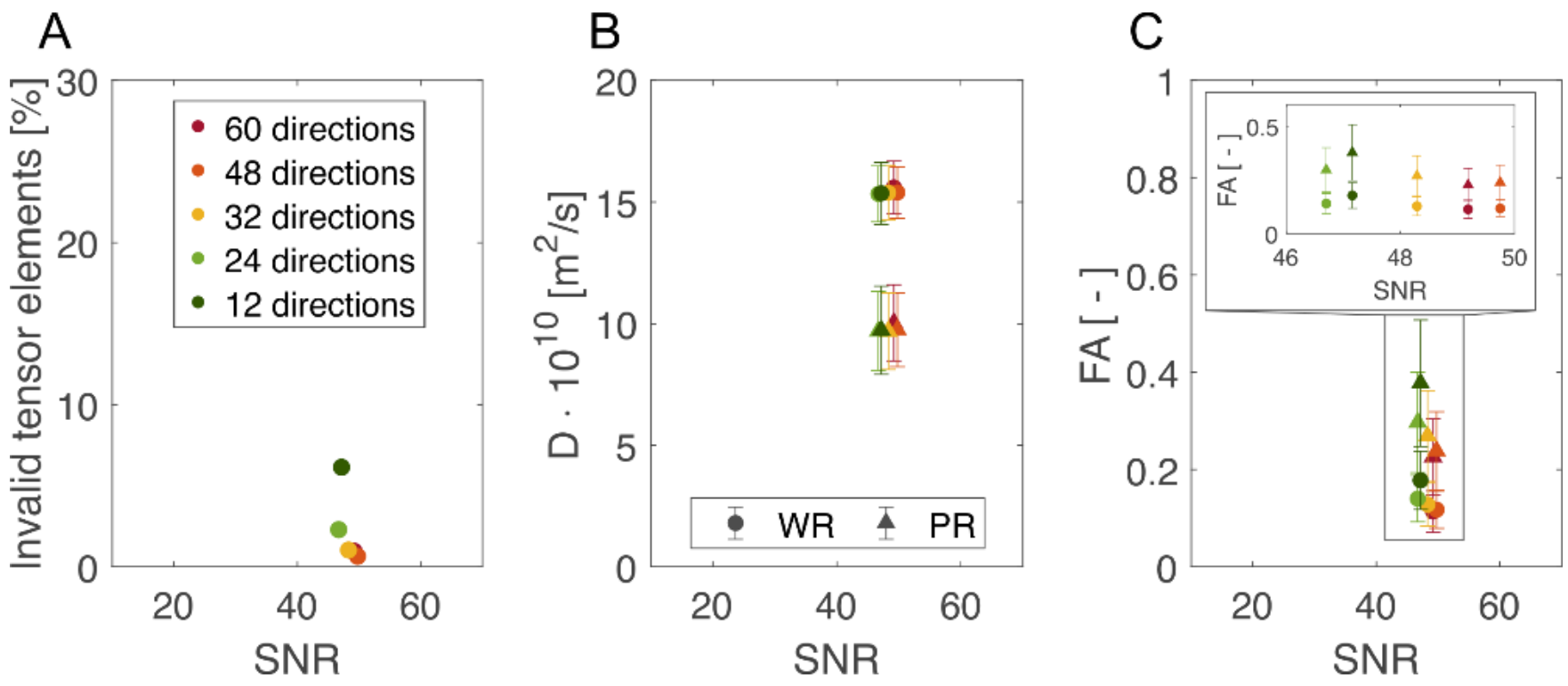

Fig. S1.5: *DT-MRI results acquired on a pH 7 SPC extrudate using an increasing number of diffusion directions. Data was acquired using 8 manually set A0-images at* b_{eff} *= 120 s/mm*^{2} *and between 12 and 60 images at b = 1000 s/mm*^{2}*. Acquisition parameters were as follows: STE with TE = 12 ms; TR = 1000 ms; voxel size = 78x78x1000 µm*^{3}*; δ = 2 ms and 4 averages were used. Images were masked for the background. (A) relative amount of non-physical tensor elements assessed by reviewing the number of voxels with one or more negative tensor elements. (B) Distribution of diffusion coefficients calculated from the trace of the diffusion tensors. (C) Distribution of fractional anisotropy values calculated per voxel from the tensor eigenvalues.*

S1.6 ROI-based Eigenvalue sorting

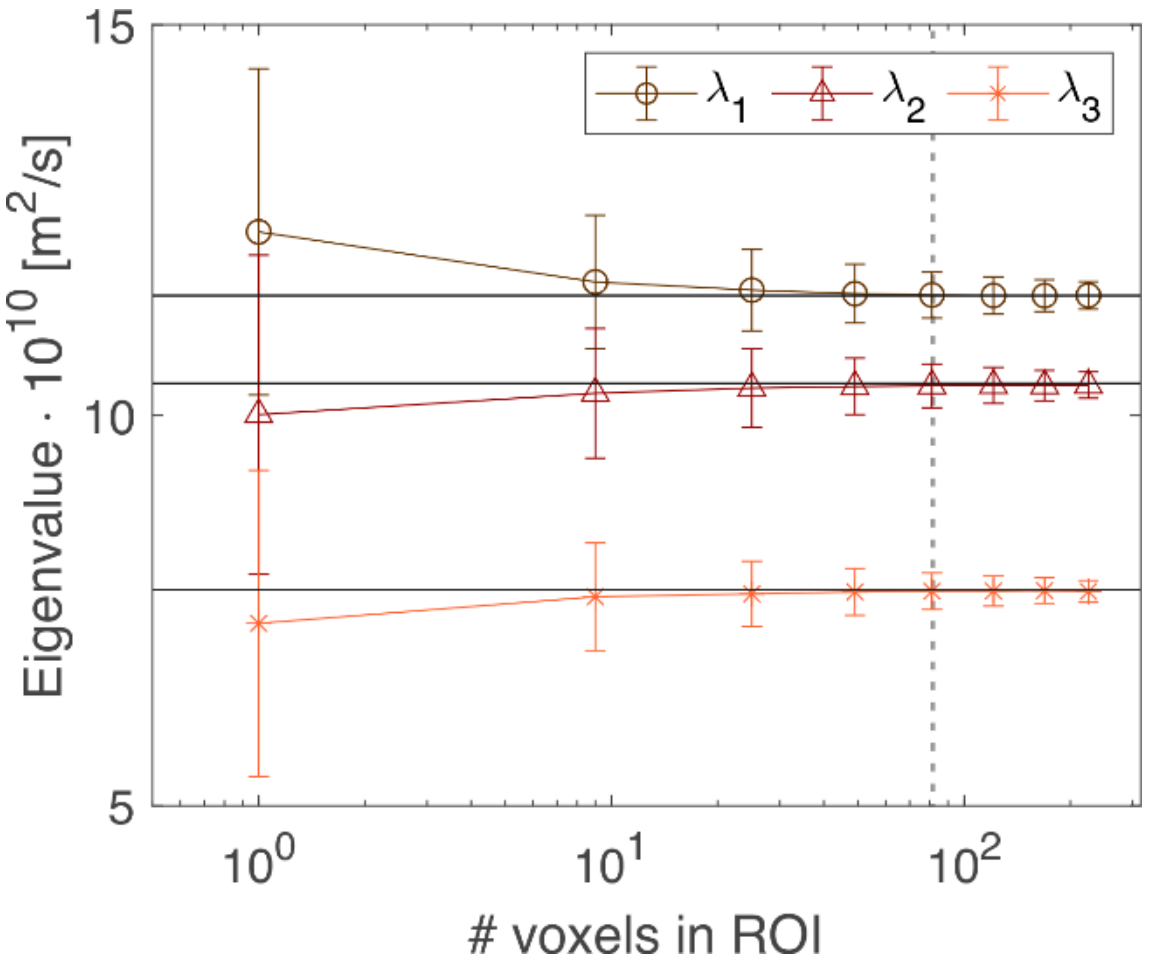

Fig. S1.5: *Mean eigenvalue distribution of ROI-based sorting for each eigenvalue (Basser* et al. *2000). The first datapoint at 10^0 voxels, i.e. a ROI of 1x1 voxel, is identical to the magnitude sorting of eigenvalues. At #voxels = 81, i.e. a 9x9 ROI region (dashed line), the eigenvalue distribution does not change with respect to ROI size. The solid lines represent the equilibrium value for the eigenvalue distribution. The error bars represent the standard error in the ROI-sorting based on ROI size, i.e. a larger ROI represents a more precise sorting. By magnitude sorting, the three eigenvalues inherently evenly distribute. However, after ROI-based sorting, the difference between the first and second eigenvalue decreases, which is expected based on the fractional anisotropy values found in these voxels of ca. 0.25.*

S2: Additional CEST and DT-MRI results

S2.1: CEST of SPI and methylcellulose dispersion

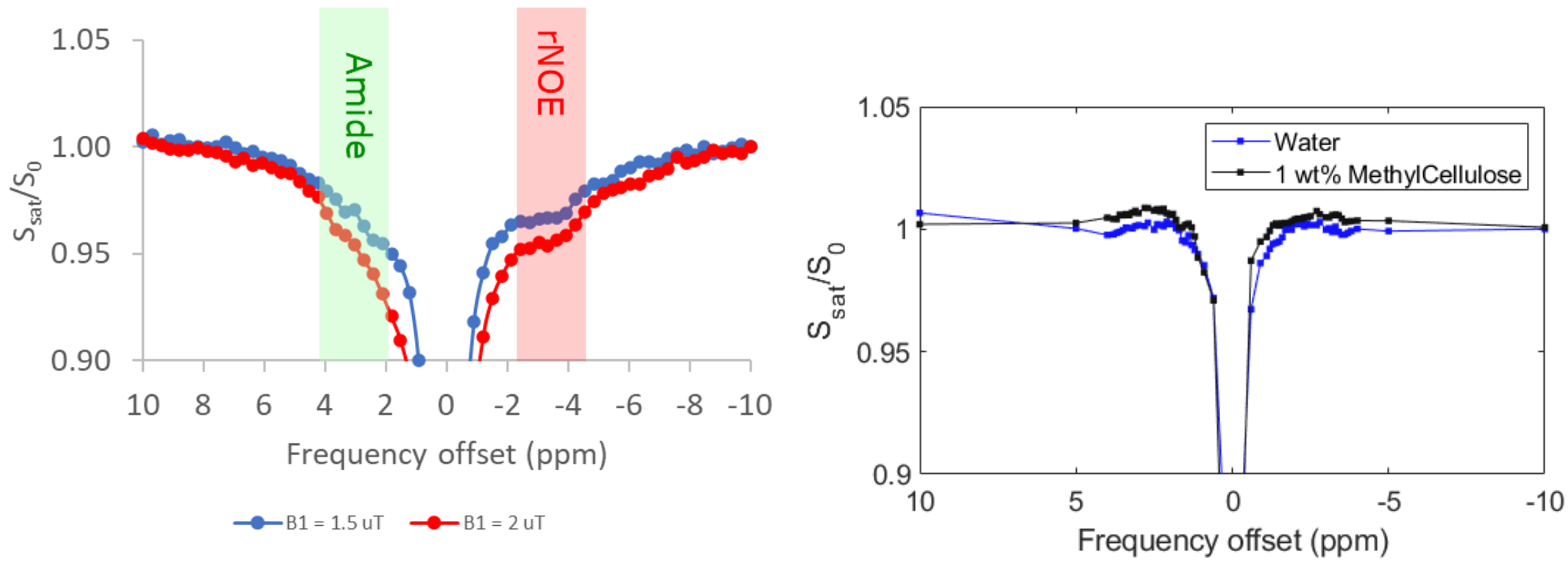

***Fig. S2.1**. CEST MRI spectra for (left) 1 wt% SPI solution and (right) 1 wt% methylcellulose solution acquired on a wide-bore 600 MHz spectrometer. In the former case measurements were conducted with T_{sat} = 1 s, B_1 = 1.5 or 2 µT, TR of 1 s, T_E = 10 ms, RARE-factor = 4, N_{avg} = 1. For data in the right, measurements were conducted using a cryo-imaging probe and T_{sat} = 1 s, B_1 = 1.5 µT, TR of 3.6 s, T_E = 5.5 ms, RARE-factor = 8, N_{avg} = 4. Only a minor peak can be observed at 1.1 ppm from the hydroxyl groups, in agreement with the expectation that methylcellulose does not produce a strong CEST effect (Han* et al. *2020).*

S2.2: CEST of soaked fresh SPC extrudate

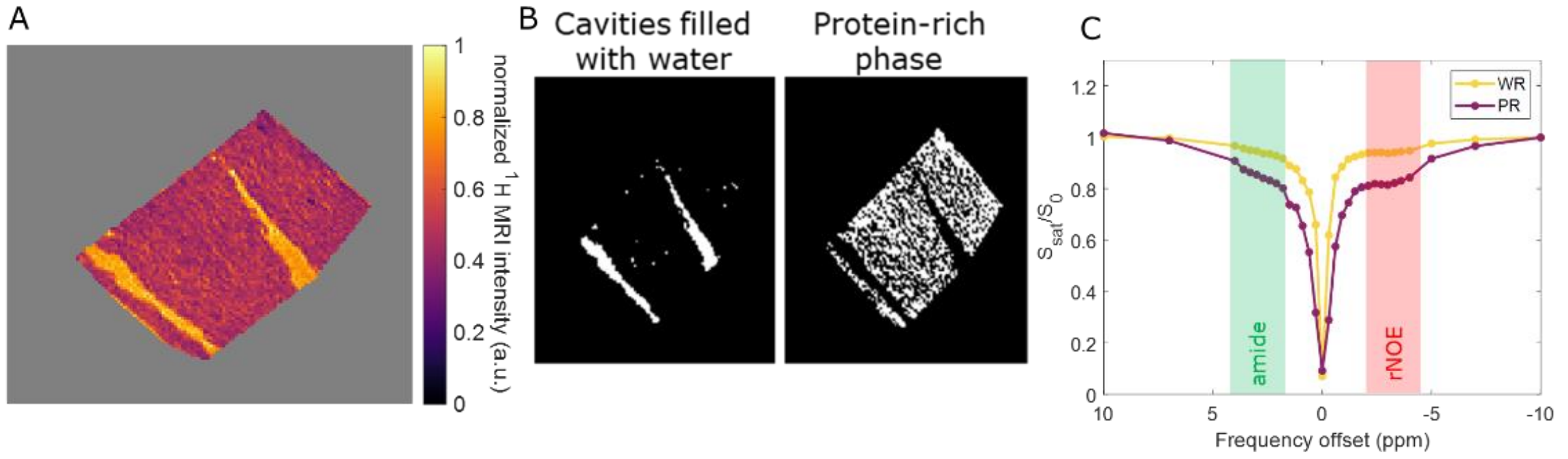

Fig. S2.2. *(A) T_2-weighted MRI image of fresh SPC extrudate from a pilot plant extruder with high ^1H MRI intensities in the cavities filled with water during soaking, and in the WR phase and low intensities in the PR phase. (B) Masks for the cavities filled with water and the PR phase. (C) CEST spectra for the water-filled cavities and the PR phase.*

S2.3: WOP histograms and statistical analysis

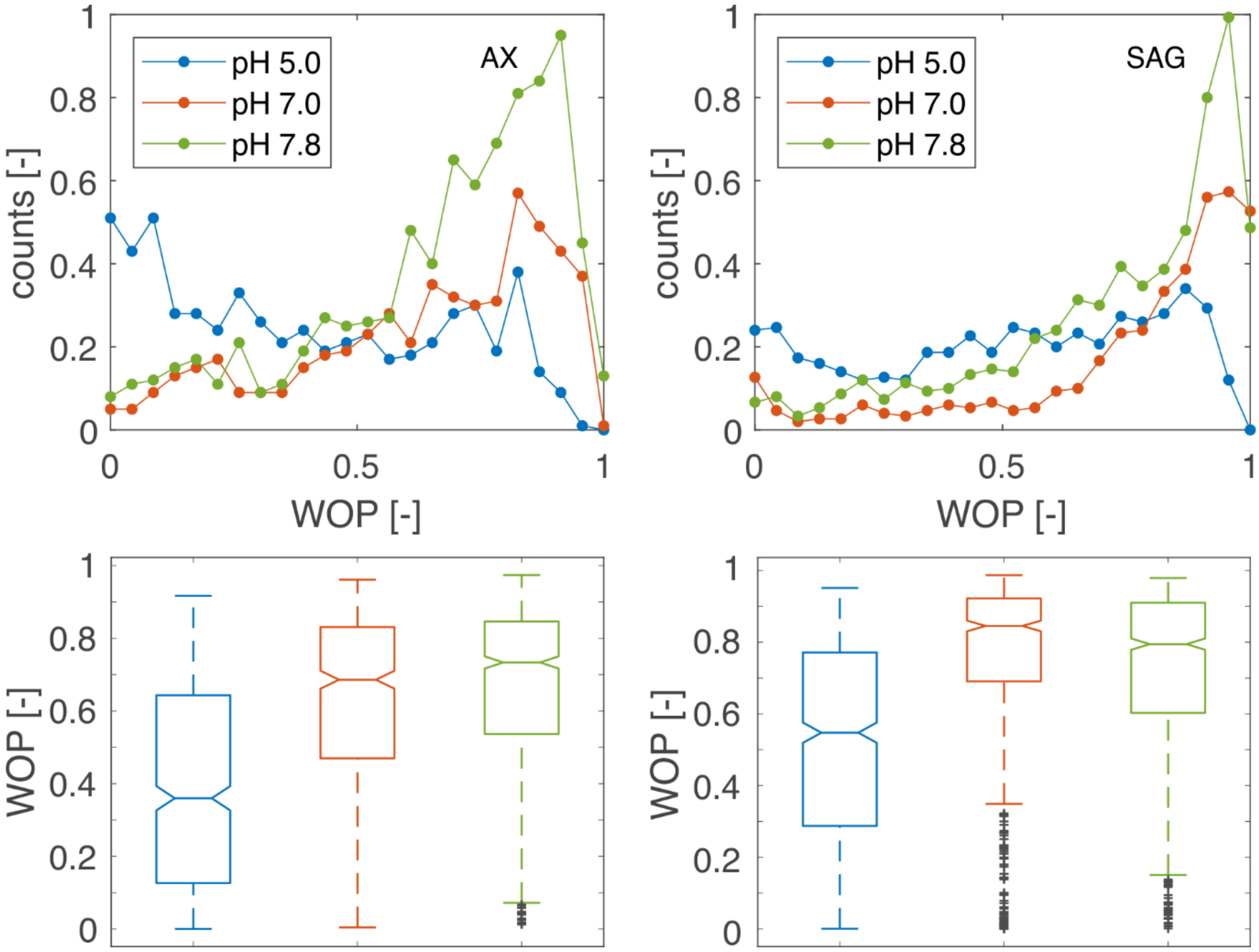

Fig. S2.3. *(Top) WOP distributions for WOP(MRI) maps presented in Fig 3B and C. For the sample at pH 7.0, the imaging region containing air bubbles was discarded from the analysis. (Bottom) box-whisker plots of WOP(MRI) distributions. ANOVA was performed for both the axial and sagittal orientation, both resulting in* $p < 0.01$*.*

S2.4: Histograms of DT-MRI diffusion and FA maps

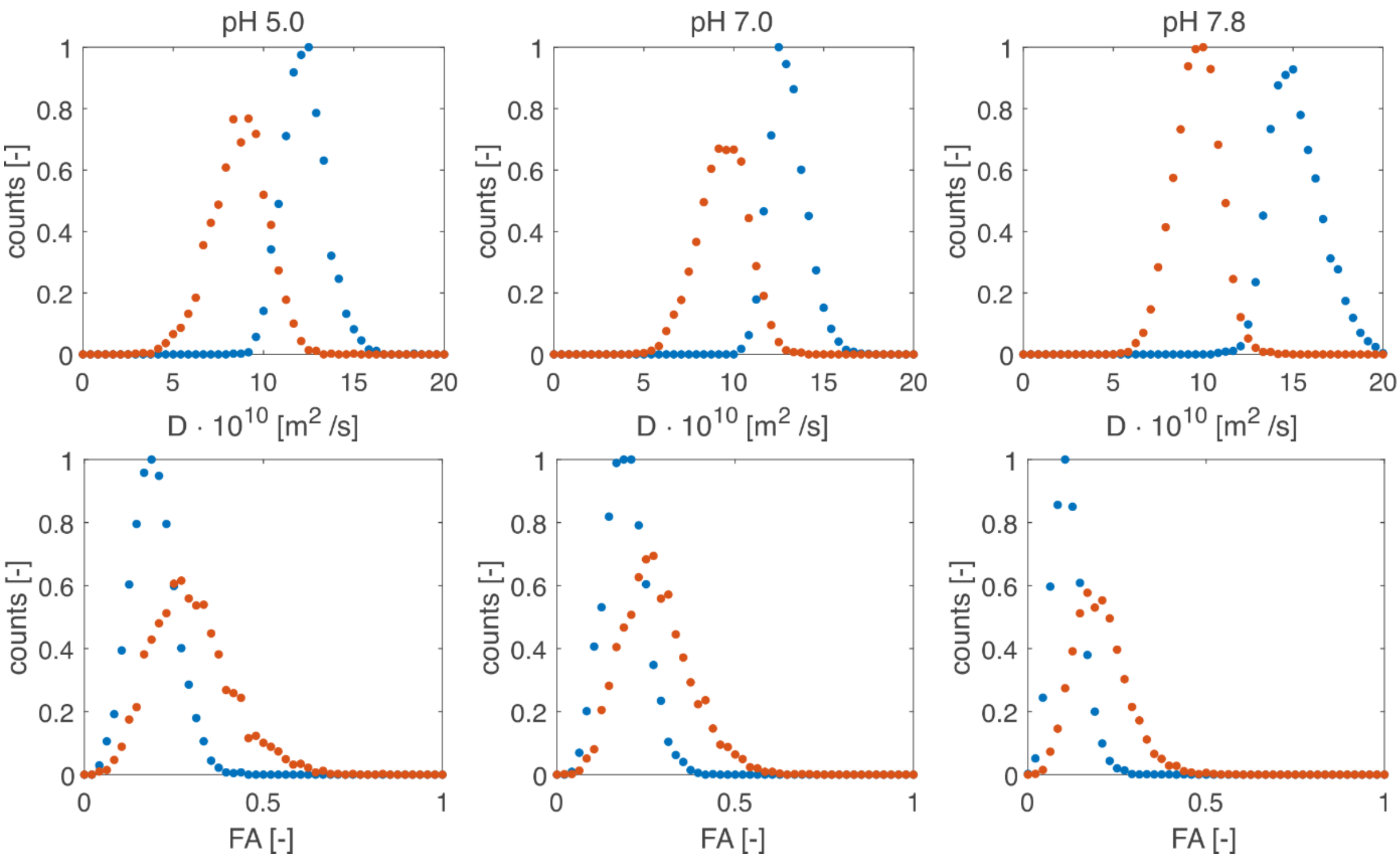

Fig. S2.4. *For the pH-shifted SPC extrudates examined by MRI in Fig. 4 of the main text, (top) self-diffusion coefficient distributions for DT-MRI maps presented in Fig 4A and (Bottom) Fractional Anisotropy distributions for FA-maps presented in Fig. 4B. Blue denotes the WR phase, orange the PR phase. Due the large difference in number of voxels in the PR phase (~50000) compared to the WR phase (~5000), a random selection of 5000 voxels from the PR phase was used to create equal sample sizes. Analysis was carried out by a two-sample Student's t-test yielded a p-value of < 0.01 for each of the six pairs of distributions.*

S2.5: MRI and DT-MRI of pilot-scale SPC extrudate

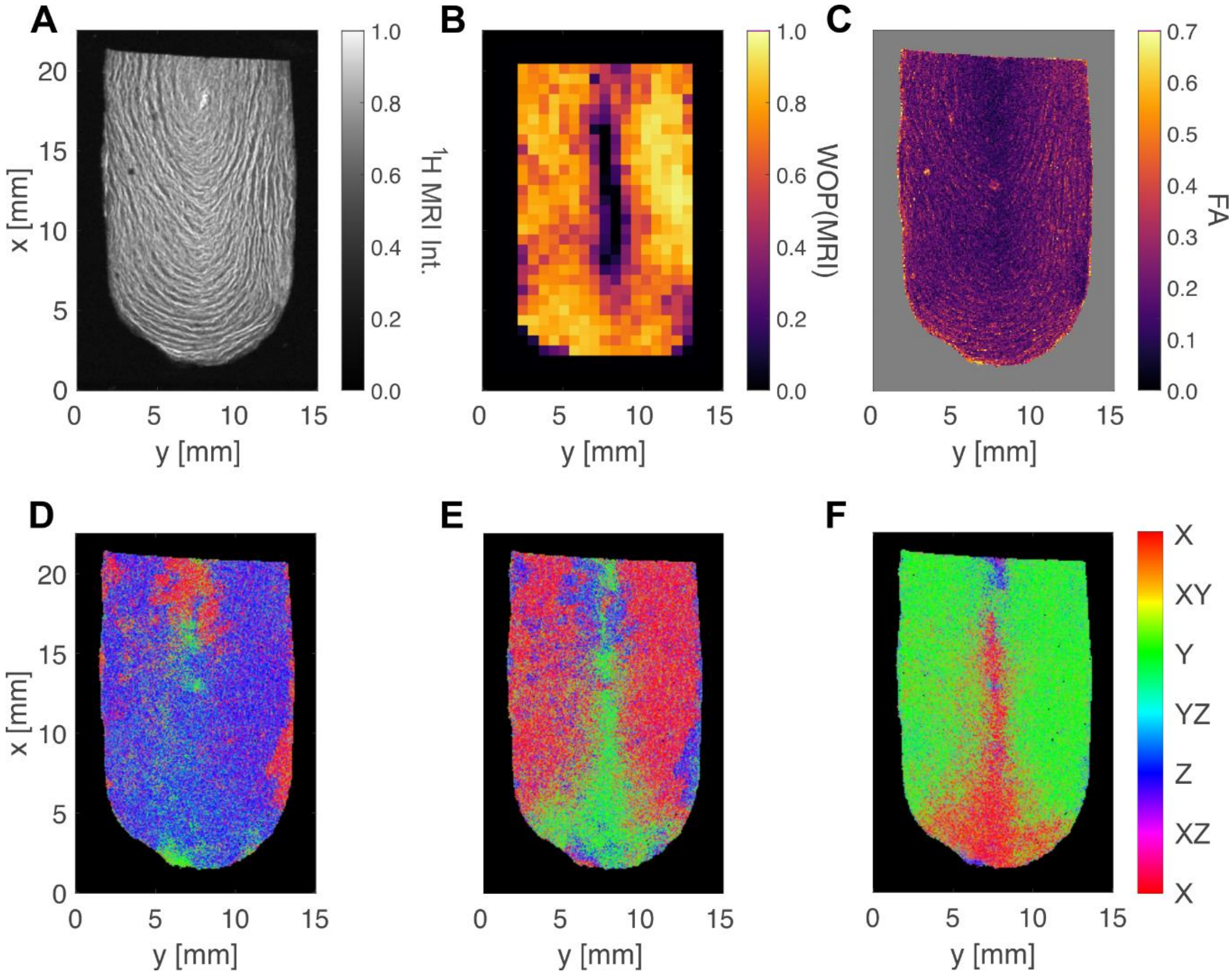

Fig. S2.3: *For SPC extrudate, shifted to pH 7, produced at pilot-scale using a round edged cooling die: T_2-weighted image (A) and respective WOP(MRI) map (B), along with DT-MRI results (C-F). Processing temperature and moisture content are similar to the extrudate samples analysed in the main text. Data was acquired using 8 manually set A_0-images at b_{eff} = 120 s/mm^2 and 36 images at b = 1000 s/mm^2. Acquisition parameters were as follows: STE with T_E = 12 ms; T_R = 1000 ms; voxel size = 78x78x1000 µm^3; δ = 2 ms and 4 averages were used. (A) T_2-weighted MRI image acquired at b_{eff} = 120 s/mm^2 showing the phase-separated structure in PR and WR phases (B) RFT WOP map of MRI image in (A), analysed using a 31 pixel window and 75% overlap, showing a mostly uniform WOP value as the structure orientation changes in the rounded corner. (C) FA map showing similar values compared to lab-scale produced SPC extrudate samples. PDD map of (D) first eigenvalue (E) second eigenvalue and (F) third eigenvalue. Eigenvalues are sorted using the ROI-based sorting established in Basser et al. (2000) using an ROI size of 9x9 voxels. Interestingly, as predicted by the modelling results of Fig. 3.5 in the main text, the dominant microstructural alignment more gradually evolves from the Y- to X-direction using a rounded die edge.*

S2.6: Mean WOP areas of synthetic cross-sectional cooling die data

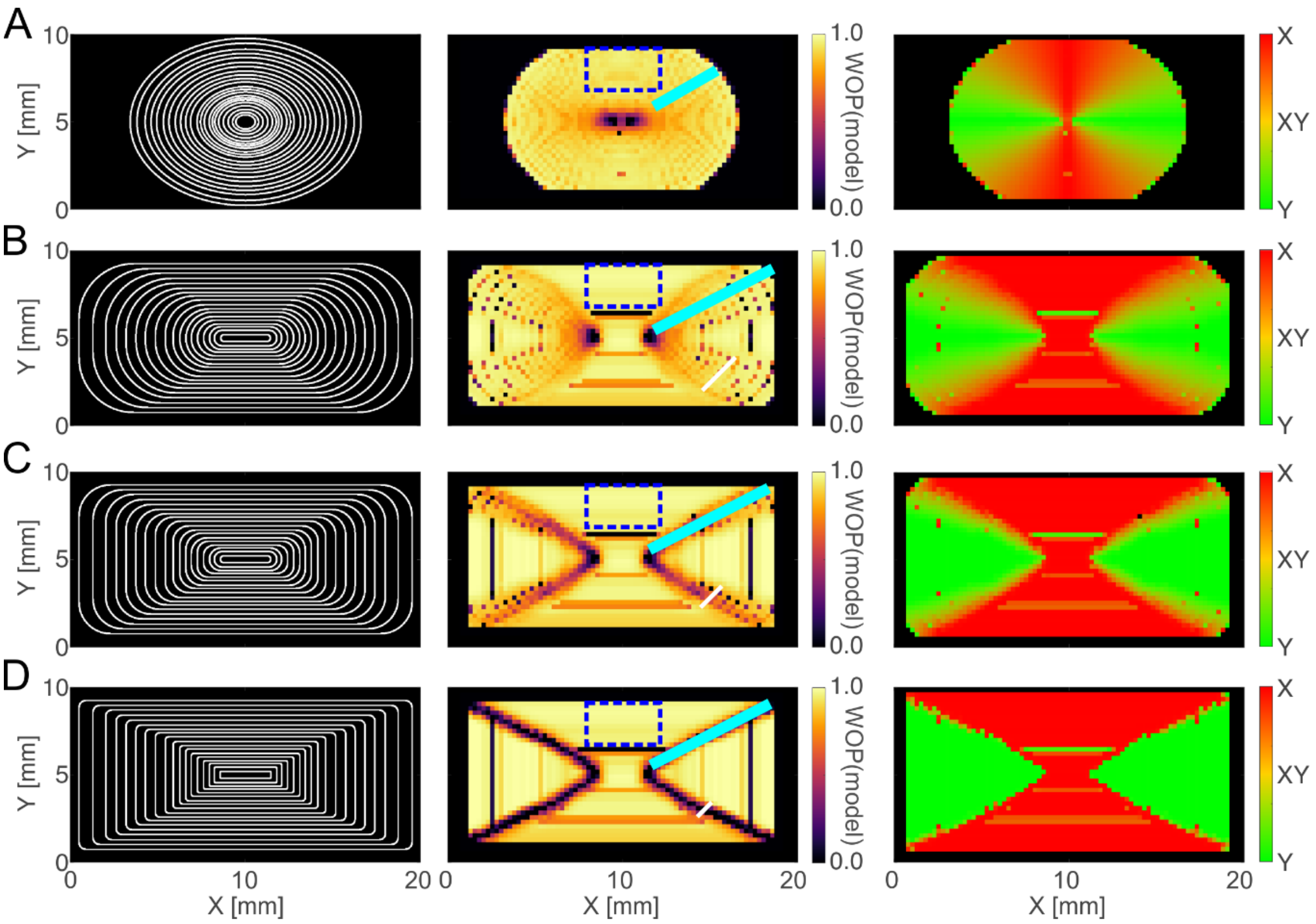

Fig. S2.4: *Synthetic axial images (left), mimicking the lamellar phase separation in SPC extrudates as seen by axial MRI data Fig. 3.3C, obtained for cross-sectional cooling die geometries with increasingly sharper corners ranging from elliptical (top) to rectangular (bottom) shaped. The synthetic MRI data, generated as described in section 2.7 of the main text, was further analysed by the RFT algorithm to create a vector field (data not shown). This vector field was used to calculate the corresponding WOP(model) (middle) and PDD(model) (right) maps. The square outlined area in dark blue shows the region of high order horizontally aligned WOP(model) values. The cyan line outlines the area used to calculate the mean WOP(model) values in the corner regions. The white line shows the location and length of the width of the corner regions as presented in section 3.3 of the main text.*

S3 Nano-scale structure assessment of mozzarella cheese

S3.1 SANS data of mozzarella cheese

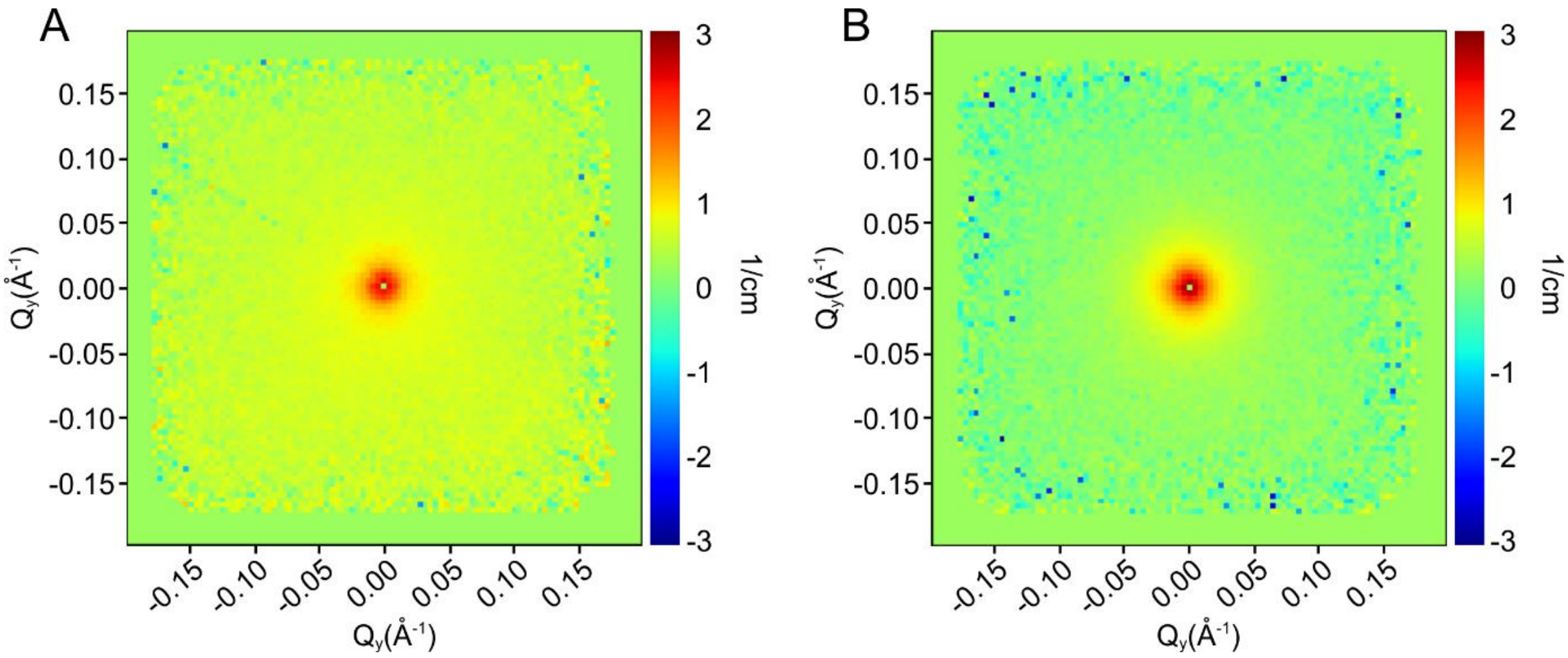

Fig. S3.1: *SANS results for a commercial high-moisture mozzarella sample. The time-of-flight LOQ instrument at ISIS Neutron and Muon source (STFC Rutherford Appleton Laboratory, UK) was used. Following standard procedures, raw data was treated to yield normalised scattering intensities I(Q) in* cm^{-1}*, where the momentum transfer is related to the scattering angle,* ϑ*, by* $Q = (4\pi/\lambda)\sin(\vartheta/2)$ *. The observed Q range was 0.007−0.23* $Å^{-1}$*. The samples were loaded into gel cell sample holders with demountable quartz windows and had a nominal thickness of 1 mm. The* D_2O *samples was soaked for ~1 hour prior to measurement. Data was reduced using the Mantid framework (Arnold* et al. *2014). (A) The 2D scattering profile acquired without contrast enhancement and (B) with contrast enhancement by swelling the sample in* D_2O *(Garina* et al. *2024). In both 2D scattering plots an isotropic pattern is observed, indicating that no significant alignment is present at length scales below 125 nm.*

References:

Basser, P. J., & Pajevic, S. (2000). Statistical artifacts in diffusion tensor MRI (DT-MRI) caused by background noise. Magnetic Resonance in Medicine, 44(1), 41–50. doi.org/10.1002/1522-2594(200007)44:1<41::AID-MRM8>3.0.CO;2-O

Han, X., Huang, J., To, A. K. W., Lai, J. H. C., Xiao, P., Wu, E. X., Xu, J., & Chan, K. W. Y. (2020). CEST MRI detectable liposomal hydrogels for multiparametric monitoring in the brain at 3T. Theranostics, 10(5), 2215–2228. https://doi.org/10.7150/thno.40146

Kingsley, P. B. (2006a). Introduction to diffusion tensor imaging mathematics: Part I. Tensors, rotations, and eigenvectors. Concepts in Magnetic Resonance Part A: Bridging Education and Research, 28, 101–122. doi:10.1002/cmr.a.20048.306

Kingsley, P. B. (2006b). Introduction to diffusion tensor imaging mathematics: Part II. Anisotropy, diffusion-weighting factors, and gradient encoding schemes. Concepts in Magnetic Resonance Part A: Bridging Education and Research, 28, 123–154. doi:10.1002/cmr.a.20049.

Kingsley, P. B. (2006c). Introduction to diffusion tensor imaging mathematics: Part III. Tensor calculation, noise, simulations, and optimization. Concepts in Magnetic Resonance Part A: Bridging Education and Research, 28, 155–179. doi:10.1002/cmr.a.20050.

Kuijpers, S. A., Goudappel, G.-J., Huppertz, T., Van Duynhoven, J. P., & Terenzi, C. (2024). Quantification of phase separation in high moisture soy protein extrudates by NMR and MRI. Food Research International, 197, 115225. doi:10.1016/j.foodres.2024.115225.

Arnold, O., Bilheux, J. C., Borreguero, J. M., Buts, A., Campbell, S. I., Chapon, L., Doucet, M., Draper, N., Ferraz Leal, R., Gigg, M. A., Lynch, V. E., Markvardsen, A., Mikkelson, D. J., Mikkelson, R. L., Miller, R., Palmen, K., Parker, P., Passos, G., Perring, T. G., … Zikovsky, J. (2014). Mantid - Data analysis and visualization package for neutron scattering and μ SR experiments. Nuclear Instruments and Methods in Physics Research, Section A: Accelerators, Spectrometers, Detectors and Associated Equipment, 764, 156–166. https://doi.org/10.1016/j.nima.2014.07.029

Garina, E. D., Adel, R. Den, Duynhoven, J. P. M. Van, Smith, G. N., Dalgliesh, R. M., Sztucki, M., & Bouwman, W. G. (2024). SANS and SAXS : A Love Story to unravel structural evolution of soy proteins and polysaccharide fibres during high moisture extrusion for meat alternatives. Food Hydrocolloids, 155, 110121. https://doi.org/10.1016/j.foodhyd.2024.110121
